# Distinctive roles of mPFC subregions in forming impressions and guiding social interaction based on others’ social behaviour

**DOI:** 10.1093/scan/nsac037

**Published:** 2022-05-17

**Authors:** Gahyun Lim, Hackjin Kim

**Affiliations:** Laboratory of Social and Decision Neuroscience, Korea University, Seoul 02841, Republic of Korea; School of Psychology, Korea University, Seoul 02841, Republic of Korea; Laboratory of Social and Decision Neuroscience, Korea University, Seoul 02841, Republic of Korea; School of Psychology, Korea University, Seoul 02841, Republic of Korea

**Keywords:** medial prefrontal cortex, cingulate cortex, decision time, social decision-making, ultimatum game, warmth

## Abstract

People can quickly form impressions of others from their social behaviour, which can guide their future social interactions. This study investigated how the type and timing of others’ social decisions affect the impression formation and social interactions. In each trial, participants watched a responder’s decision in an ultimatum game, decided whether to choose the responder as their next partner for proposer or responder and reported the perceived warmth, competence and likability of the responder. Participants preferred responders who accepted (i.e. accepters) unfair offers for the responder and those who rejected (i.e. rejecters) unfair offers for the proposer in their next ultimatum game, and the rostral medial prefrontal cortex (mPFC) activity encoded such a strategic context-dependent valuation when choosing partners. Slow rejecters were perceived as warmer than fast rejecters, which was mirrored by the anterior mid-cingulate cortex activity when watching others’ decisions, possibly detecting and resolving conflicting impressions. Finally, those who perceived accepters *vs* rejecters as warmer showed higher ventral mPFC responses to accepters *vs* rejecters when choosing a partner, regardless of the context. The present study suggests that distinctive subregions of the mPFC may be differentially involved in forming impressions and guiding social interactions with others based on their social behaviours.

## Introduction

An accurate estimation of others’ social preferences is essential for successful social interactions because it can help predict whether someone would be harmful or helpful to oneself (Fiske *et al.*, 2007; Liberman *et al.*, 2014). The ability to infer others’ social preferences develops at an early stage in humans, even in infancy (Hamlin *et al.*, 2007; Fawcett and Liszkowski, 2012; Hamlin, 2013; Lee *et al.*, 2015), and is closely related to various psychiatric disorders (King-Casas and Chiu, 2012) including autism spectrum disorder (Yoshida *et al.*, 2010; Chambon *et al.*, 2017), schizophrenia (Chambon *et al.*, 2011) and social anxiety (Hirsch and Clark, 2004; Blair *et al.*, 2010).

A common way of estimating others’ social preferences is to observe their previous choices in social contexts. People often form social impressions of strangers based on their previous decisions, which then guides future interactions. For instance, third-party observers are likely to punish those who had treated others unfairly and reward those who had treated others fairly (Nowak and Sigmund, 1998; Wedekind and Milinski, 2000; Leimar and Hammerstein, 2001; Fehr and Fischbacher, 2004). The previous choices of decision-makers influence the likelihood of receiving the shares of others’ resources or being chosen as a partner in the future (Nowak *et al.*, 2000; Horita, 2010; Ozono and Watabe, 2012; Raihani and Bshary, 2015).

However, the choice *per se* may not be sufficient to precisely predict others’ social preferences or personality traits, because people often adjust their behaviours deliberately in socially desirable ways (Rand *et al.*, 2012, 2014; Rand, 2016). Thus, people might consider various information to form the other’s impression, including how fast they decide and what their goals are, in addition to what they choose. In particular, the speed of one’s social decision-making provides crucial information for estimating their social preferences (Rand *et al.*, 2012; Rand, 2016; Yamagishi *et al.*, 2017), because it may reflect the strength of their relative preference for the chosen option (Evans and Stanovich, 2013; Krajbich *et al.*, 2015; Konovalov and Krajbich, 2018). Recent studies have shown that people consider others’ decision time in forming their impressions or planning social interactions with them. For example, quick immoral/selfish behaviour strengthens the negative evaluations of the actor, compared to the same but slower behaviour (Critcher *et al.*, 2013; Evans and van de Calseyde, 2017). Moreover, a partner’s decision time for cooperation can influence one’s future economic engagement with them (Van de Calseyde *et al.*, 2014; Evans and Rand, 2019) and the level of trust toward them in subsequent trust games (Jordan *et al.*, 2016b). Also, the goal or context of social decision-making could modulate the process of planning social interactions based on the social impressions of strangers. For example, people perceived the person who had contributed a large amount of resources in the common asset as helpful when the goal of the contribution was cooperation (e.g. a public good game), but not when it was an investment (e.g. risk-taking game) (Cooper *et al.*, 2010). Moreover, people asymmetrically updated the impression when observing the social *vs* non-social agents (Boorman *et al.*, 2013). Or people modulated the subjective value of a prosocial product which elicited more buying behaviour under social observation (Jung *et al.*, 2018).

The medial prefrontal cortex (MPFC) has been strongly implicated in social impression formation (Mitchell *et al.*, 2004, 2005, 2006; Van Overwalle, 2009; Cloutier *et al.*, 2011; Mende-Siedlecki *et al.*, 2013; Ferrari *et al.*, 2016a, 2016b) and value computation for social decisions (Van Den Bos *et al.*, 2007; Hare *et al.*, 2010; Corradi-Dell’Acqua *et al.*, 2013; Civai *et al.*, 2014; Ruff and Fehr, 2014; Sul *et al.*, 2015; Jung *et al.*, 2018). The mPFC contains multiple anatomically and functionally heterogeneous subregions (Bzdok *et al.*, 2013; De La Vega *et al.*, 2016; Lieberman *et al.*, 2019; Kim, 2020), consisting of at least three parts: the ventral (vmPFC), rostral (rmPFC) and dorsal mPFC (dmPFC). According to a recent model of the mPFC’s function in allostatic regulation (Kim, 2020), the subregions of the mPFC are organised hierarchically along the ventral-to-dorsal axis such that the vmPFC and the dmPFC are involved in intuitive internal valuation and deliberative external valuation, respectively, and the rmPFC is involved in strategic modulation of internal valuation according to external situational constraints (Jung *et al.*, 2018; Yoon *et al.*, 2018, 2021; Kim and Kim, 2021).

**Fig. 1.**
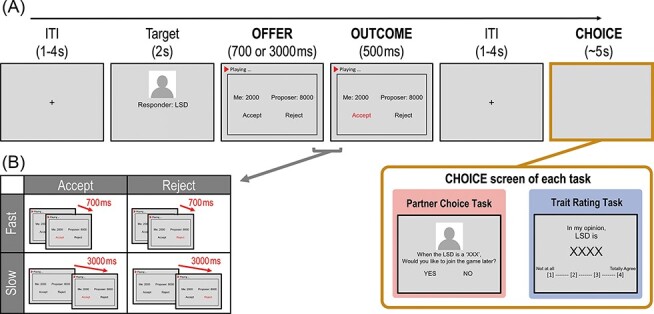
Task design and experimental conditions. (A) The task screen of the ‘partner choice task’ and the ‘trait rating task’. In both tasks, a participant viewed a responder (of the ultimatum game)’s initial ID (Target screen) and their ostensibly recorded video clip which consisted of the offer the responder had received (OFFER screen) and the decision they had made (OUTCOME screen). The time interval between the OFFER screen and the OUTCOME screen manipulated the responder’s type. One of the CHOICE screens appeared depending on the task following the cross screen which distinguished the responders’ recorded screen and the participant’s choice screen. The participant chose whether they want to be the partner of the video clip’s responder who would be the future proposer or a responder depending on the trial in the ‘partner choice task’ or rate how much the responder would be likely to possess the personality traits (warmth, competence and likability) in ‘trait rating task’. (B) Experimental conditions of the responder type. The responders of the ultimatum game were suggested within five types in the video clips. The responder decided to reject (rejecter condition) or accept (accepter condition) in a short time (700 ms; fast condition) or after a relatively long time (3000 ms; slow condition) subsequent to the unfair offer. Therefore, we had four experimental conditions: fast rejecter, slow rejecter, fast accepter and slow accepter. In addition, we included the control condition in which the responder quickly accepted the fair offer (fast accepter).

Thus, this study investigated (i) how the type and time of others’ social decision-making affect impression formation and (ii) how such information modulates the observer’s social interactions with the observed person as the goal of decision-making varies, using functional magnetic resonance imaging (fMRI) in the context of the ultimatum game (Güth *et al.*, 1982; Camerer, 2003; Sanfey *et al.*, 2003). In each trial, participants watched a video clip showing a responder’s decision ostensibly recorded during a previous ultimatum game; they accepted or rejected the unfair offer either quickly or slowly (Figure 1). Participants were then prompted to decide whether they chose the responder as their own proposer or responder in the next ultimatum game (Task 1). Upon completion, participants watched the eight example trials of the same video clips again (i.e. two for each condition) outside the MRI machine and reported the perceived warmth and competence which are the two crucial dimensions of social cognition (Fiske *et al.*, 2002, 2007; Cuddy *et al.*, 2008) known to influence social decision-making (Holoien and Fiske, 2013; Bratanova *et al.*, 2015; Rudert *et al.*, 2017; Azevedo *et al.*, 2018) and the responder’s likability (Task 2).

We hypothesised that decision time and type would differentially influence impression formation measured by warmth and competence and that the context of the decision would prioritise the type of information to be considered and influence the subsequent valuation of partners. Based on theoretical and empirical studies, we predicted that distinctive mPFC subregions would be differentially engaged in impression formation and partner choice, depending on the social information derived from others’ social behaviours.

## Methods

### Participants

Forty-four participants (16 women, mean age = 23.7 years, age range = 20–30) were recruited from Korea University’s online community. In the behavioural and fMRI data analyses, we excluded one participant due to abnormal visual acuity and three participants who misunderstood the instructions or structure of the experiment. In the fMRI data analyses, one participant who fell asleep during the fMRI task was excluded. Thus, the final fMRI analysis included 39 participants (15 women, mean age = 23.8 years, age range = 20–30). All participants included in the data analyses were healthy and right-handed, without any history of mental disorders. All participants provided informed consent, and the experimental protocol was approved by the Institutional Review Board of Korea University. The participants received Korean Won (KRW) 40 000 (US dollar (USD) 40) as a participation fee, KRW 37 500 for the fMRI and behavioural experiments and an additional monetary incentive of KRW 2500 for the pre-task ultimatum game and surveys.

### Task and procedures

#### Overview of the experimental procedures

This experiment consisted of three behavioural tasks: (i) the pre-task ultimatum game, (ii) the partner choice task for the next ultimatum game (main task) and (iii) the trait rating task. Participants were informed that they would participate in two ultimatum games over two visits and that, on the first visit, they would be the responder and select the partner pool in the partner choice task for the second visit. Participants performed the three tasks consecutively and performed the partner choice task inside the scanner. They revisited the laboratory and completed surveys on a separate day (Supplementary Text 1.5.). All participants were debriefed and paid within 3 weeks of participation.

#### Pre-task ultimatum game

Participants were asked to perform the pre-task ultimatum game prior to the partner choice task. This task was to familiarise participants with the structure of the ultimatum game and with the perspectives of responders. Moreover, it was to make the participants believe the video clips used in the main task were from real participants. In the pre-task ultimatum game, a proposer determines the division of KRW 10 000 (USD 10) between the proposer and responder in each trial. Both can earn the amounts suggested by the proposer if the responder accepts the monetary offer. On the other hand, neither can earn money if the responder rejects it. All participants performed 10 trials as responders. They were told that all monetary offers had been suggested by prior participants acting as proposers. Unlike the typical ultimatum game, however, we simplified the monetary offers using only two ratios, 8:2 and 5:5, representing unfair and fair offers, respectively. Out of 10 trials, six were unfair and four were fair. There were more unfair trials than fair trials since we were only interested in the unfair condition. But, the ratio of unfair:fair conditions were set at 6:4 to prevent the participants from expecting the fair proposers to be highly rare and salient. The participants were told that the result of one randomly chosen trial would be given as an incentive. The detailed procedures are shown in Supplementary Figure S1.

#### Partner choice task for ultimatum game

In each trial of the partner choice task, participants first saw a video clip of ostensibly recorded screens from a previous responder of the ultimatum game and then were asked to decide whether they would choose the responder as their potential partner in a future ultimatum game. The video clips contained a proposer’s offer amount [fair (5:5) or unfair (8:2)], followed by a responder’s decision (accept or reject). The decision time of the responder was manipulated by the duration between the offer and decision outcome events. It was intended to make the participants perceive the fast or slow decision in a more ecologically valid manner and to manipulate different conditions implicitly without explicitly requiring the participants to focus on the decision time to avoid a potential demand characteristic. In support of this idea, the debriefing data showed that no participants had precisely guessed the objectives of our study with our task. The decision time was set at either 700 ms (i.e. fast decision) or 3000 ms (i.e. slow decision), based on the data obtained from an independent study which was used to identify the decision times that most people consistently perceive as either fast or slow decision (see Supplementary Text 1.3). Participants were informed that their partners for the second ultimatum game would be randomly chosen from the responders they had chosen in this task. We designed our task to prevent the participants from being influenced by the prior decisions of the same target; therefore, each target of the partner choice task was identified as a unique initial and presented only once.

The unfair conditions were further divided into four sub-conditions: fast accepter, fast rejecter, slow accepter and slow rejecter conditions. The task also included 30 trials of accepting fair (i.e. 5:5) offer quickly (i.e. fair accepter) to make the participants believe the offers are from the real proposer, but these trials were not used in the behavioural data analysis. Participants were asked to choose their partner for a proposer in half of the trials and a responder in the other half. Each of these 10 conditions (i.e. 5 responder conditions × 2 roles) was presented 15 times in the main task, resulting in a total of 150 trials.

Each trial started with the initials of the responder (2 s) in the video clip. Next, the video clip displayed the OFFER screen with a monetary offer for the responder and the option to ‘Accept’ or ‘Reject’. It was followed by the OUTCOME screen, where the chosen option turned red after 700 ms or 3000 ms, depending on the condition. The OUTCOME screen lasted 500 ms, followed by the fixation cross screen (1–4 s) to distinguish the video clip from the participant’s own choice (CHOICE screen). On the CHOICE screen, the question ‘When XXX (responder’s initials) is a “proposer” (or “responder”), would you like to join the game later?’ with the options ‘YES’ and ‘NO’ displayed below (Figure 1, Task 1). To make a choice, participants used a four-button magnetic resonance (MR)-compatible response grip to press the first button with their index finger or the second button with their middle finger. Their chosen option then turned red for 500 ms, and the fixation screen with a crosshair was shown for 1–4 s.

#### The trait rating task

In the trait rating task, participants were instructed to judge the responders for warmth and competence, the two fundamental dimensions of social perception (Fiske *et al.*, 2002, 2007; Cuddy *et al.*, 2008), as well as for likability using a four-point Likert scale (1: ‘not at all’ to 4: ‘totally agree’) outside the scanner (Figure 1, Task 2). The three traits were shown successively in random order for each video clip. Participants were told that the video clips of the trait rating task were not included in the partner choice task. The trait rating task consisted of eight trials, and only four types of the responder who received unfair offers were presented. Each type was presented twice.

## Analyses

### Behavioural data analyses

To examine the influences of decision type, decision time and partner’s role in the partner choice task, we executed a 2 (role of partner: proposer or responder) × 2 (decision type: accept or reject) × 2 (decision time: slow or fast) three-way repeated-measures analysis of variance (ANOVA) on the mean percentage of partner choices and the reaction time. Moreover, we also checked the effect of the type and time of the responder within each partner role using a 2 (decision type: accept or reject) × 2 (decision time: slow or fast) two-way repeated-measures ANOVA (rmANOVA) on the partner choice and reaction time data separately for the proposer and responder choice conditions. The reaction time data were standardized across all trials within each participant.

For the trait rating task, we hypothesised that decision type and time would impact the perception of social traits. Thus, a 2 (decision type: accept or reject) × 2 (decision time: slow or fast) two-way rmANOVA was implemented on the warmth, competence and likability ratings.

We also investigated whether the influence of warmth and competence ratings on partner choices would differ depending on the partner’s role by employing a generalised linear mixed model (GLMM) on the binary responses of the partner choices (0: No and 1: Yes), using the ‘glmer’ function of the ‘lme4’ (version 1.1-23) package in R studio (version 1.2.5033). The model included fixed-effect predictors of the partner’s role (1: proposer and 2: responder) as a categorical variable, the rating scores of perceived warmth and competence of each responder as numerical variables and their interaction variable, as well as the random effects of each participant accounting for the effect of the intercept, the warmth and competence score and the interaction of the warmth and competence score. The perceived rating scores of warmth and competence were mean-centred such that the range changed from 1–4 to −1.5–1.5.

We ran additional exploratory data analyses to examine the perceived ambiguity of each responder’s impression (see Supplementary Text 3) and the temporal effects of decision type and time on the partner choice (see Supplementary Text 4 and Supplementary Table S1).

### fMRI data processing and analyses

The information about fMRI data acquisition is reported in Supplementary Text 2.2. The first-level general linear model included the onsets of OFFER screen events, OUTCOME screen events and CHOICE events. In OFFER onset, the onsets of unfair offers and fair offers were included as separate regressors. We divided the OUTCOME onsets into five regressors: FastRejecter, SlowRejecter, FastAccepter, SlowAccepter and FairAccepter. The choice onset comprised 10 regressors of five types of responders separately for the proposer and responder choice conditions. The neural activities with respect to the OFFER onset were modelled using a canonical haemodynamic response function (HRF) convolved with a boxcar function for the duration of the OFFER screen depending on the fast or slow condition (3000 ms or 700 ms), while the neural activities with respect to the OUTCOME or CHOICE onsets were modelled by a canonical HRF convolved with a stick function at the onset time. In addition, we applied additional GLM that was similar to the main GLM but included the trial-by-trial reaction time data as a parametric modulator of the CHOICE onset regressor (Supplementary Figure S4). The reaction time data were standardised across all trials within each participant.

Based on the behavioural results, we aimed to find the neural correlates related to two interaction effects: (i) the interaction between decision type and decision time when a participant observed the responder’s decision and (ii) the interaction between the partner’s role and the decision type when a participant chose the responder as their proposer or responder. First, for the decision type × decision time interaction effect, the first-level contrast maps of ‘[SlowRejecter—FastRejecter]—[SlowAccepter—FastAccepter]’ at the OUTCOME onset were created and entered into the second-level one-sample *t*-test. Second, for the role × decision type interaction, the first-level contrast maps of ‘Proposer [Rejecter—Accepter]—Responder [Rejecter—Accepter]’ at the CHOICE onset were created and entered into the second-level one-sample *t*-test.

In addition to our main analyses, we explored the neural activations which might reflect the individual differences in the subjective warmth perceptions between the rejecters and the accepters. Considering the higher warmth ratings and partner choice rates for the accepters compared to the rejecters, the influence of decision type on the warmth perception might involve neural activities which are associated with the processing of values. We focused on the subregions of mPFC that are highly related to value computation and social decision-making (Kable and Glimcher, 2007; Kim *et al.*, 2007; Bartra *et al.*, 2013; Kim, 2020). The subjective differences in warmth perception were calculated by subtracting the warmth ratings of the rejecters from those of the accepters (i.e. ‘accepter warmth—rejecter warmth’). The warmth ratings of the four responders were normalised within each participant. This score regressed the contrast map of ‘Accepter—Rejecter’ of the OUTCOME and CHOICE onset. The resulting maps of these multiple regressions were thresholded the small volume correction using the binary mask map of mPFC which defined the subregions from the functional coactivation map (De La Vega *et al.*, 2016).

In the behavioural and fMRI data analyses, we excluded trials in which the participants missed any part of the video clip to ensure that full information about the responders was available for impression formation. For details on the procedure for excluding trials, see Supplementary Text 2.2.

The neuroimaging results reported in this study were thresholded at *P* < 0.05 and corrected for the peak-level family-wise error (FWE) rates or the cluster-level FWE which were defined at the initial uncorrected *P* < 0.001.

## Results

### Behavioural results

#### Trait perception according to decision type and time

To investigate the effect of decision type and time on the trait perception of the ultimatum game responder, we ran a 2 (decision type: accept or reject) × 2 (decision time: fast or slow) rmANOVA on the ratings of warmth, competence and likability.

**Fig. 2.**
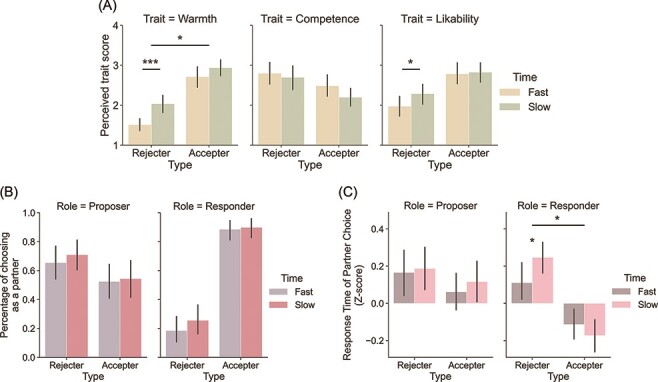
Behavioural results of the trait rating task. (A) The mean scores of trait ratings: warmth (left), competence (middle) and likability (right). (B) The mean percentages of partner choice: proposer (left) and responder (right). (C) The mean scores of standardised RT for the partner choice: Proposer (left) and Responder (right). The error bars indicate the 95% confidence interval.

For warmth perception, the main effects of decision type [*F*(1, 39) = 77.21, *P* < 0.01] and decision time [*F*(1, 39) = 15.40, *P* < 0.001] were significant (Figure 2A, left). Participants perceived accepters as warmer than rejecters (*M*_Accept_ = 2.83, SD_Accept_ = 0.80, *M*_Reject_ = 1.78, SD_Reject_ = 0.68) and slow responders warmer than fast responders (*M*_Slow_ = 2.49, SD_Slow_ = 0.84, *M*_Fast_ = 2.11, SD_Fast_ = 0.94). The interaction effect of decision type and time was also significant [*F*(1, 39) = 4.54, *P* < 0.05], showing a higher degree of difference in perceived warmth between slow and fast responders compared to accepters. *Post hoc* analyses showed that slow rejecters were perceived as significantly warmer than fast rejecters [*t*(39) = 4.48, *P* < 0.001], but the difference between slow and fast accepters was not statistically significant [*t*(39) = 1.88, *P* = 0.068].

Regarding competence perception, participants perceived rejecters (*M*_Reject_ = 2.75, SD_Reject_ = 0.88) as marginally more competent than accepters [*M*_Accept_ = 2.34, SD_Accept_ = 0.74; *F*(1, 39) = 3.57, *P* = 0.066] (Figure 2A, middle).

For likability perception, participants liked accepters (*M*_Accept_ = 2.82, SD_Accept_ = 0.81) more than rejecters [*M*_Reject_ = 2.13, SD_Rejecter_ = 0.84; *F*(1, 39) = 19.01, *P* < 0.001] (Figure 2A, right).

#### Effects of partner’s role and decision type on partner choice

To examine the effects of responder’s decision type and time on partner choice, we ran a 2 (partner’s role: proposer or responder) × 2 (decision type: accept or reject) × 2 (decision time: fast or slow) rmANOVA. This analysis showed a significant interaction effect of the partner’s role and decision type: *F*(1, 38) = 62.32, *P* < 0.001 (Figure 2B). *Post hoc* analyses revealed that participants chose accepters (*M*_Accept_ = 0.89, SD_Accept_ = 0.22) more than rejecters (*M*_Reject_ = 0.22, SD_Reject_ = 0.30) as their responder: *t*(38) = 10.019, *P* < 0.001. They showed a tendency to choose rejecters (*M*_Reject_ = 0.68, SD_Reject_ = 0.35) over accepters (*M*_Accept_ = 0.53, SD_Accept_ = 0.41) as their proposer, although this effect was not statistically significant [*t*(38) = −1.47, *P* = 0.15]. In addition, there was a significant main effect of decision type, *F*(1, 38) = 15.25, *P* < 0.001, which showed that participants chose the accepter more than the rejecter. However, there were no other main effects [Role: *F*(1, 38) = 2.58, *P* = 0.12; Decision time: *F*(1, 38) = 2.68, *P* = 0.11] or interaction effects [Role × Decision Time: *F*(1, 38) = 0.02, *P* = 0.90; Decision Type × Decision Time: *F*(1, 38) = 1.31, *P* = 0.26; Role × Decision Type × Decision Time: *F*(1, 38) = 0.32, *P* = 0.58]. Importantly, we note that participant’s choices were not affected by the decision time of the responders.

In addition, we explored whether the null effect of the decision time was caused by the participant’s learning that the decision time information was not predictive where the structure of the task had orthogonalised the decision type and time. In that case, the effect of decision time on partner choice might have been significant in the early trials but decreased over time. We divided the unfair trials (i.e. 120 trials) into three blocks of 40 trials (i.e. 2 roles × 4 unfair offer responders × 5 repetitions) as the early, mid- and late periods of the task. Then we investigated whether the blocks influenced the decision time effect or its interaction with decision type with the 2 (role: proposer or responder) × 2 (type: rejecter or accepter) × 2 (time: fast or slow) repeated-measure analysis of covariance with the covariate of block numbers (1, 2 and 3) (Supplementary Text 4). However, the block did not influence any effects of the decision time or its interaction effects (Supplementary Table S1). Also, we checked the 2 (type: rejecter or accepter) × 2 (time: fast or slow) rmANOVA on each role of each block (Supplementary Text 4). The time main effect on the responder choice was not significant in any of the three blocks. Meanwhile, the time main effect on the ‘proposer’ choice seemed to, rather, increase in block 3 [*F*(1, 37) = 4.491, *P* = 0.041], but not in block 1 or 2 [block 1: *F*(1, 38) = 0.004, *P* = 0.949; block 2: *F*(1,38) = 1.767, *P* = 0.192] (Supplementary Table S1 and Supplementary Figure S2).

#### Effects of partner’s role and decision type on the reaction time of partner choice

We ran a 2 (partner’s role: proposer or responder) × 2 (decision type: accept or reject) × 2 (decision time: fast or slow) rmANOVA on the standardised reaction time (RT)s of partner choices. This analysis showed a significant main effect of role [*F*(1, 38) = 5.25, *P* < 0.05], decision type [*F*(1, 38) = 22.46, *P* < 0.001] and a significant role × decision type interaction effect: *F*(1, 38) = 7.12, *P* < 0.05 (Figure 2C). Participants took more time to choose their proposers compared to responders (*M*_proposer_ = 0.13, SD_proposer_ = 0.36; *M*_responder_ = 0.02, SD_responder_ = 0.33) and to consider the rejecters as their partner compared to the accepters (*M*_rejecter_ = 0.18, SD_rejecter_ = 0.34; *M*_accepter_ = −0.03, SD_accepter_ = 0.33). There was a trend of role × decision type × decision time interaction effect [*F*(1, 38) = 3.68, *P* = 0.063], but it was not statistically significant. In addition, a 2 (decision type: accept or reject) × 2 (decision time: fast or slow) rmANOVA was conducted on the RTs of partner choices separately for the proposer and responder choice conditions. In the responder choice, we found a significant main effect of decision type [*F*(1, 38) = 37.36, *P* < 0.001] and a decision type × decision time interaction effect: *F*(1, 38) = 7.96, *P* < 0.01. *Post hoc* analyses revealed that participants spent more time considering slow rejecters (*M*_slowrejecter_ = 0.24, SD_slowrejecter_ = 0.28) than fast rejecters (*M*_fastrejecter_ = 0.11, SD_fastrejecter_ = 0.31): *t*(38) = 2.32, *P* = 0.026. On the other hand, the difference in RT between slow and fast accepters was not significant: *t*(38) = −1.35, *P* = 0.186. No significant main or interaction effect was found in the RT of proposer’s choice.

#### The relationship between perceived traits and partner choice

Next, we performed a GLMM to investigate whether participants weighted the two traits (i.e. warmth and competence) differently for partner choice depending on the role. The analysis showed a significant interaction effect between the role and warmth ratings (*b* = 1.61, SE = 0.10, *X*^2^(1) = 269.31, *P* < 0.001), such that the influence of perceived warmth on partner choice was greater in the responder than the proposer condition. In addition, the interaction effect between the role and competence rating was significant (*b* = −0.59, SE = 0.10, *X*^2^(1) = 40.63, *P* < 0.001), such that the influence of perceived competence on partner choice was greater in the proposer than the responder condition. The results of the other fixed and random effects are listed in Supplementary Table S2.

### fMRI results

#### Neural correlates of the interaction effect of decision type and decision time

**Fig. 3.**
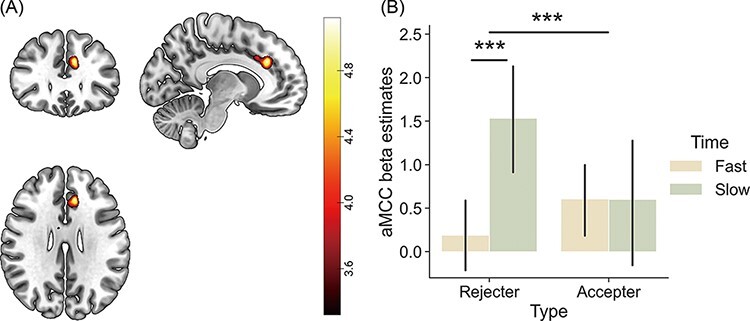
The fMRI results from the decision type × decision time interaction effect at the OUTCOME onset. (A) The brain activation map of the decision type × decision time interaction on the OUTCOME onset. The cluster of aMCC (peak coordinates: *x *= −10, *y *= 30, *z *= 28) differently reflected the influence of a responder’s decision time for the rejecter and the accepter when encoding a responder’s decision outcome. (B) The bar graph of beta estimates from the aMCC ROI. After having watched the decision outcome of a rejecter, the aMCC activated more when the decision was slow compared to when it was fast. After having watched the accepter’s outcome, however, there was no difference between the slow and the fast condition in the aMCC activation. The activation maps of figures are illustrated in uncorrected *P* < 0.001 results.

In parallel with the behavioural results, we investigated the neural correlates encoding the interaction effect of decision type and time when observing the responder’s decision. The group contrast map of the decision type × time interaction ([SlowRejecter − FastRejecter] − [SlowAccepter − FastAccepter]) at the OUTCOME onset revealed significant activation of the anterior midcingulate cortex (aMCC: *x* = −10, *y* = 30, *z* = 28; *P* = 0.028, FWE cluster-level corrected) (Figure 3A). The magnitude of aMCC activity was higher when observing the decision of a slow rejecter compared to a fast rejecter [*t*(38) = −4.71, *P *< 0.001], whereas the difference in the aMCC activation between fast and slow accepters was not significant: *t*(38) = 0.014, *P* = 0.989 (Figure 3B). The opposite contrast (i.e. [FastRejecter − SlowRejecter] − [FastAccepter − SlowAccepter]) showed no significant cluster of activity.

#### Neural response to the interaction of partner’s role and decision type

**Fig. 4.**
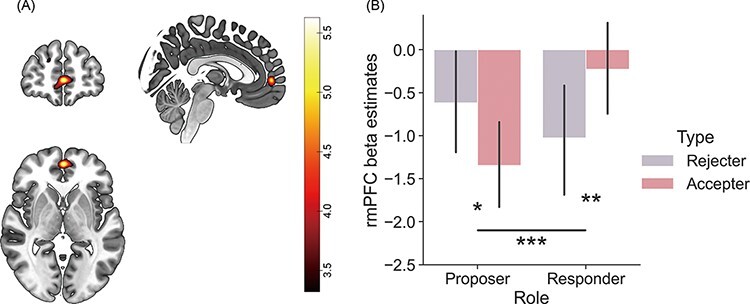
The fMRI results from the decision type × role interaction effect at the CHOICE onset. (A) The brain activation map of the decision type × partner role interaction on the CHOICE onset. The activation of rmPFC (peak coordinates: *x* = 0, *y* = 54, *z* = 0) showed the interaction effect between the potential role of the partner and the decision type when the participant chose the responder as a future partner. (B) The bar graph of beta estimates from the rmPFC ROI. The activation of rmPFC decreased when the participant considered an accepter, compared to a rejecter, as a future proposer. On the contrary, the activation of rmPFC increased when considering an accepter, compared to a rejecter, as a future responder. The activation maps of figures are illustrated in uncorrected *P* < 0.001 results.

Next, in parallel with the behavioural results, we examined the neural correlates encoding the interaction effect of the partner’s role and decision type during the partner choices. A second-level one-sample *t*-test on the individual contrast maps of the role × type interaction ([proposer (Rejecter − Accepter) − responder (Rejecter − Accepter)]) at the CHOICE onset showed a significant activation cluster in the rmPFC (*x* = 0, *y* = 54, *z* = 0; *P* = 0.034, FWE peak-level corrected) (Figure 4A). In line with the behavioural results, the magnitude of rmPFC activity was higher when considering the accepter, compared to the rejecter for the responder [*t*(38) = −2.859, *P* = 0.007], whereas the pattern was reversed when considering the proposer [*t*(38) = 2.603, *P* = 0.013] (Figure 4B). On the other hand, one may argue that this activity could reflect the differences in decision time among conditions, particularly given that the interaction effect of the role and decision type on the reaction time was also significant. To address this issue, we ran another GLM that was similar to the main GLM but included the reaction time data as a parameter modulator of the CHOICE onset regressor to control for potential RT effects. In this analysis, the peak activation of the rmPFC cluster decreased (*Z* = 4.36, *P*_peak-FWE_ = 0.163) but was still significant at the cluster-level threshold (*P*_cluster-FWE_ = 0.010; Supplementary Figure S4). This might imply that the rmPFC contributed to the context-dependent valuation of partner choices, even after controlling for the reaction time differences. The opposite contrast (i.e. [proposer (Accepter − Rejecter) − responder (Accepter − Rejecter)]) showed no significant cluster of activity.

#### Neural response correlating the difference in warmth perception depending on the decision type

**Fig. 5.**
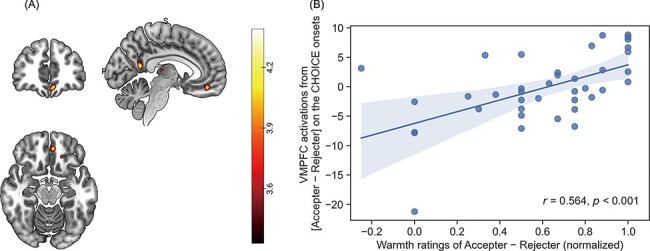
The vmPFC activity encoding idiosyncratic perceived warmth of accepters *vs* rejecters when choosing a partner for the ultimatum game. (A) The neural activations of the VMPFC (peak coordinates: *x *= −6, *y *= 44, *z *= −16) were positively correlated with the normalised warmth ratings of ‘accepters—rejecters’ when the participants had to choose their partners (SVC corrected, *P*_SVC-clusterFWE_ = 0.049, cluster size = 82). (B) The scatterplot of the VMPFC activations and the subjective differences in the warmth ratings between the accepters and the rejecters. The shaded area of this graph indicates the 95% confidence interval. The activation maps of this figure are illustrated in uncorrected *P* < 0.001 results.

Given that participants behaviourally differentiated the rejecters and the accepters in the perception, the value related to the warmth rating might be represented in the neural area. We explored whether the subregions of mPFC, which are related to the value computation and social decision-making, might be correlated with the subjective differences in warmth ratings between the rejecters and accepters. We searched the neural area which was correlated with the individual differences in warmth perception between accepter and rejecter on the corresponding contrast maps (i.e. Accepter − Rejecter) of both OUTCOME and CHOICE onset within mPFC region of interest (ROI) (De La Vega *et al.*, 2016). It could not find any significant correlation in OUTCOME onset. On the other hand, the vmPFC cluster (peak coordinates: x = −6, y = 44, z = −16, small volume correction (SVC) corrected, *P*_SVC-clusterFWE_ = 0.049, cluster size = 82) of the accepter *vs* rejecter was positively correlated with the warmth differences of the accepter and the rejecter on the CHOICE onset (Figure 5), which might imply that the warmth valuation of the responder might be represented in this area independent of the contextual changes.

## Discussion

This study investigated whether and how the decision type and time of responders in the ultimatum game influence the formation of their impressions and the likelihood of being chosen as a partner for future social interactions. As expected, when observing the responders’ decisions, participants considered not only the decision type but also the decision time for impression formation, such that accepters were perceived to be warmer than rejecters and slow responders to be warmer than fast responders. In addition, fast responders were perceived to be less warm than slow responders only when they rejected unfair offers, but not when they accepted them. A similar differential effect of the decision time depending on decision type was also found on the dmPFC (i.e. aMCC) activity when watching the responders’ decisions. In addition, participants’ preferences for the responder’s decision type varied depending on the decision context, such that they were more likely to choose accepters as their own ‘responder’ partners and to choose rejecters as their own ‘proposer’ partners. Such a behavioural pattern was in parallel with the neural activation pattern of the rmPFC when participants chose their future partners in the ultimatum game. Finally, those who perceived accepters as warmer than rejecters showed higher vmPFC responses to accepters *vs* rejecters at the partner choice phase, regardless of the context. The present findings suggest that observing others’ social decisions can affect the formation of impressions and subsequent social interactions with them, and distinctive subregions of the MPFC are differentially involved in these processes.

Our experimental design allowed participants to be indirectly exposed to others’ decision times which presented in the video clips as the duration of others’ decision-making, instead of making them read specific numbers of decision speed or hypothetical scenarios (Critcher *et al.*, 2013; Van de Calseyde *et al.*, 2014; Jordan *et al.*, 2016a; Evans and van de Calseyde, 2017). This could prevent explicit information about decision time to convey that such information is important and worth considering which might lead to potential task demand characteristics (Orne, 1962; Nichols and Maner, 2008; Nichols and Edlund, 2015). As predicted, the present study demonstrated that decision time modulated the perceived warmth and likability of the responders even when it was revealed indirectly through behaviours.

Why did decision time influence the evaluation of rejecting, but not accepting responders? One interpretation is that social acceptance and rejection are hard-wired with more fundamental psychological mechanisms of approach and avoidance systems, respectively. Supporting this idea, the acceptance rate of unfair offers was modulated by the induced motivation of approach-avoidance (Harlé and Sanfey, 2010). Also, the social perception of warmth trait is associated with the approach-avoidance motivation. These associations might be automatically activated in the process of social perception (Peeters, 2002; Freddi *et al.*, 2014) because it could provide essential information for friend-or-foe judgements critical to survival (Fiske *et al.*, 2007). Consistent with the well-known behavioural strategy of ‘win-stay, lose-shift’ (Nowak and Sigmund, 1993), avoidance may employ a greater degree of environmental exploration than approach (Kim, 2020). Just as avoidance requires switching the current strategy with additional information seeking, estimating the impression of a rejecting responder may increase the demand for seeking additional information, such as decision time.

The aMCC was the only brain region showing a significant decision type × time interaction when participants observed the decisions of others. In parallel with the behavioural results, the aMCC showed the most prominent activation in response to slow rejecters. The aMCC is anatomically homologous to the dorsal ACC, which has been strongly implicated in monitoring response conflicts (Botvinick *et al.*, 2004; Holroyd and Yeung, 2012; Ebitz and Hayden, 2016), and is also part of a larger anatomical region called the dmPFC. According to recent theories, the dmPFC is engaged to accumulate additional external information from the surrounding environment when a conflict of values must be resolved (Kim, 2020) and to calculate the need for energy or persistent behaviours to achieve a goal by integrating multimodal sensorimotor information (Touroutoglou *et al.*, 2020). Therefore, aMCC activation in the present study may reflect the conflict or ambiguity associated with the valuation of the responder, signalling the increased need for additional external information from the environment to resolve the conflict. Given that the aMCC was activated predominantly in the slow rejecter condition, it can be inferred that participants experienced a greater conflict; therefore, they required additional external information such as decision time to estimate the impression of the slow rejector compared to all other responders (Hofmann and Bitran, 2007; Kim, 2020).

There is evidence that slow rejectors elicited stronger conflict in impression formation. First, the perceived warmth and likeability of a slow decision-maker seem to be the opposite of that of a rejecter. The choice disposition of the partner choice appeared to be different between the rejecter and accepter and between slow and fast decision-makers. For example, participants chose the accepter as their partner more often than the rejecter on average, particularly in the responder choice condition, which required relatively easier and more intuitive decisions than the proposer choice condition, as shown by the significant decision type × time interaction in the reaction time. However, participants also perceived slower decision-makers as warmer and more likeable than fast decision-makers. Thus, we can infer that forming an impression of slow rejecters can be trickier than those of others because information on decision type and time conflict with each other in the formation of perceived warmth. Why did the aMCC activity increase for slow rejecters but not for fast accepters? As mentioned above, consistent with the ‘win-stay, lose-shift’ strategy, people may be more motivated to seek additional information in the case of avoidance (i.e. rejecter) *vs* approach (i.e. accepter), leading to more consideration of decision time when forming an impression of a rejecter than an accepter. Second, a slow rejecter is perceived to be closer to the indifference points in trait evaluations and partner choices. For example, the mean likability ratings of slow rejecters were not statistically different from the midpoint of the scale, and the percentage of partner choice of the slow rejecter was not different from the chance level (50%). Third, participants showed the longest reaction time for slow rejecters in the partner choice task, possibly indicating the highest level of conflict or ambiguity in the evaluation of slow rejecters. In summary, the increased aMCC activity in response to slow rejecters may reflect an increased demand for additional information to resolve conflicts in trait evaluation and subsequent partner choice.

We found no effect of decision time on either the percentage of partner choice or neural activation at the time of partner choice. Given that more obvious social signals can shunt attention away from less obvious ones (Jordan *et al.*, 2016a), the null effect of decision time may have been at least partly because participants placed greater weight on decision type than decision time, because the former is more obvious information than the latter. Alternatively, responders’ decision time may have indirectly influenced the partner choices by adjusting the perceived traits of potential partners. The effect of each social trait on partner choice differed depending on the role of the partner, such that participants placed higher weight on perceived warmth and lower weight on perceived competence when choosing responders compared to when choosing proposers, which was independent of the experimental condition of each responder in the video clip. Given that the role of the partner was not revealed to participants until the CHOICE screen, it could be inferred that the aMCC contributed to the adjustment of trait impression of the responders based on decision type and time, and participants then used those trait impressions for subsequent partner choices, flexibly changing the weights of different traits depending on the suggested role of the partner. On the other hand, there is another alternative hypothesis that it was because the participants had got to believe that decision time information is negligible for the partner choice after learning that the decision time and the decision type were orthogonalised. However, it seems unconvincing given that the experimental time (i.e. block) did not influence any effects involving the decision time. Rather, the decision time effect on the proposer choice had increased in the late period. This might imply that participants may have paid more attention to the decision time information as they had gained abundant evidence for the distribution of their potential partner pool, especially in the condition where the decision type does not accurately predict the behaviour of potential partners in the future ultimatum game (i.e. proposer choice condition). Nevertheless, the influence of decision time on decision-making might have increased if the participants had performed a different task where they could be more benefitted from choosing warm or competent partners. Future studies would be necessary to test the effect of decision time in diverse contexts.

The rmPFC appears to be the key system flexibly changing the weights of different traits depending on the suggested role of the partner because the rmPFC activity at the time of partner choice mirrored the interaction effect of decision type and the role in the percentage of the partner choice. More specifically, rmPFC activity increased in response to rejecters *vs* accepters in the proposer choice condition, whereas it showed the reverse pattern of activity in the responder choice condition, revealing its role in context-dependent valuation for partner choice. This is consistent with previous empirical and theoretical studies showing that the rmPFC plays a key role in the process of allostatic regulation to adjust one’s internal bodily needs to the surrounding external environment (Kim, 2020). For example, the rmPFC differently encoded the value of monetary contribution depending on whether the contribution was described as a donation or a risky investment (Cooper *et al.*, 2010). In addition, the rmPFC contributes to strategic social behaviours to maximise social rewards depending on the context. For example, an increase in rmPFC activity is associated with increased socially desirable behaviour (Tusche *et al.*, 2016; Cutler and Campbell-Meiklejohn, 2019; Fukuda *et al.*, 2019), self-enhancement under social observation (Izuma *et al.*, 2010; Somerville *et al.*, 2013; Muller-Pinzler *et al.*, 2016; Van Hoorn *et al.*, 2016; Jung *et al.*, 2018; Yoon *et al.*, 2021) and tracking of self-efficacy or social status (Korn *et al.*, 2012; Kumaran *et al.*, 2016; Ligneul *et al.*, 2016; Wittmann *et al.*, 2016; Will *et al.*, 2017; Yoon *et al.*, 2018). In this regard, the change in rmPFC activity depending on the role of the partner could be understood as a strategic context-dependent valuation addressing the differences in the social hierarchy and/or monetary advantage between the proposer and responder in the ultimatum game.

In the present study, the preference for rejecters over accepters was only marginally significant in the proposer context, unlike the responder choice condition where participants chose accepters significantly more than rejecters. Previous studies have demonstrated that those who punish unfair behaviours by others can be preferred as future partners (Horita, 2010; Ozono and Watabe, 2012). This is at least partly because one’s punishment behaviour, such as rejecting an unfair offer in an ultimatum game or third-party punishment, might signal their trustworthiness (Jordan *et al.*, 2016a) by indicating that they would follow the rule of fairness (Nowak *et al.*, 2000; Fehr and Fischbacher, 2003), and moral condemnation can be an effective signal of one’s moral integrity (Jordan *et al.*, 2017). In contrast, punishment can also be interpreted as personal revenge or anger (Gilam *et al.*, 2015). These two competing interpretations of punishing immoral behaviours could be responsible for the inconsistency in the results of favouring a punisher as a partner (Ozono and Watabe, 2012; Raihani and Bshary, 2015; Przepiorka and Liebe, 2016) and may also provide some interpretation of the mixed results of partner choice for proposer in this study. More specifically, some participants might prefer a rejecter to an accepter as their proposer because they wanted a fair proposer, while others showed the opposite preference because the rejection behaviours reflected personal revenge or aggression. However, when considering partners for the responder, most of the participants unequivocally preferred accepters to rejecters, prioritising generosity over fairness.

On the other hand, the value difference of warmth between the rejecter and the accepter was represented in the vmPFC activation regardless of the contextual modulations on the partner choice. It has been reported that the vmPFC is engaged in the intuitive valuation compared to the dorsal subregions of mPFC (Sul *et al.*, 2015; Hackel *et al.*, 2020; Kim, 2020) and the value processing which is independent of the goal or context (Hare *et al.*, 2009; Jung *et al.*, 2018; Tusche and Hutcherson, 2018; Qu *et al.*, 2020). Judging the other’s warmth is crucial in that the warmth trait of someone could lead to their potential helpful or harmful behaviours, so it occurs rapidly to prepare the perceiver’s own tendency of approach-avoidance (Fiske *et al.*, 2007; Li *et al.*, 2021). Thus, this value might be intuitively represented in the vmPFC regardless of the contextual modulation. On the other hand, the correlation between the differences in warmth and the neural activation of vmPFC was significant on the CHOICE onset but not OUTCOME onset. In our task, participants had to incorporate the decision time and the decision type to evaluate the responders of the ultimatum game, which might require the time to compare the potential reward from the rejecter and the accepter. This could be achieved by the distinctive contribution of aMCC and vmPFC, which might respectively track the specific attributes (i.e. decision time) or the integrative value (i.e. warmth) through evidence accumulation (Shenhav *et al.*, 2018).

It is noteworthy that we could not find any evidence for tight relationships between behaviours and the neural activities of the aMCC or the RMPFC. First, as for the aMCC, its activity seems to reflect conflict processing rather than simply tracking the warmth trait itself in the formation of social impression. For example, the targets who are certainly warm (i.e. accepter) or not warm (i.e. fast rejecter) would not activate the aMCC because they do not cause conflicts. However, the target with the conflicting impression (i.e. slow rejecter) would elicit aMCC activation because rejecting the proposer’s offer and longer decision time might contradict each other when forming an impression, as evidenced by the intermediate level of warmth rating given to this target. Second, as for the rmPFC, its activity appears to reflect heightened weights on the information related to valuation for decision, not necessarily reflecting behavioural choice itself. For example, the desirable traits of potential partners could vary depending on their roles in the ultimatum game, given that the degree to which warmth and competence influenced partner choices were modulated by the role of the partner. Thus, the role × type interaction of the rmPFC activation might reflect an increased need for processing the values that are more relevant to the changed context but less intuitively considered to directly predict the actual choices. Besides, our experimental design includes only two levels of decision time (i.e. 3000 or 700 ms), which was intended to allow the participants to perceive the differences in the decision as clearly as possible, preventing the participants from perceiving identical decision times differently depending on prior sequences of decision time. However, it still remains unanswered whether and how the present findings can be generalised to more naturalistic settings with near-normal distributions of decision time.

## Conclusions

In conclusion, this study demonstrated that observation of others’ social decisions can affect the formation of impressions and subsequent social interactions with them, and distinctive subregions of the MPFC can be critically and differentially involved in these processes. The dmPFC, including the aMCC, contributed to the adjustment of trait impressions of the responders based on decision type and time, which could be used to guide subsequent partner choices. In addition, the rmPFC was engaged to flexibly change the weights of different traits depending on the suggested role of the partner, consistent with the suggested function of the rmPFC in strategic context-dependent valuation. Finally, the vmPFC showed higher responses to accepters *vs* rejecters among those who perceived accepters *vs* rejecters as warmer, regardless of context, consistent with its well-known role in intuitive value assessment. We believe that the present findings provide important clues to help us understand the psychological and neural mechanisms whereby simple observation of others’ social behaviours can lead to the formation of impressions that influence social interaction.

## Supplementary Material

nsac037_Supp

## Data Availability

The data that support the findings of this study are available on request from the corresponding author.
