# Supplementary material for: Distinctive roles of mPFC subregions in forming impressions and guiding social interaction based on others’ social behaviour
Source: Soc Cogn Affect Neurosci. 2022 May 17;17(12):1118–30. doi: 10.1093/scan/nsac037 (PMC9714428; doi:10.1093/scan/nsac037)
Supplement: nsac037_Supp [file nsac037_supp.zip › Supplementary Material.docx]

**Supplementary Material**

**Supplementary Text 1. Task and procedures**

*1.1. The overview of the experimental procedure*

This experiment consisted of three behavioural tasks: (a) the pre-task ultimatum game, (b) the partner choice task for the next ultimatum game, and (c) the trait rating task. Upon visiting the laboratory, participants were explained about the ultimatum game. Participants were also informed that they would participate in two ultimatum games over two visits and that, on the first visit, they would be the responder and select the partner pool for the second visit. Then, they performed the partner choice task in the scanner. In a trial, they watched multiple video clips of the ostensibly recorded screen from the responders of the ultimatum game and decided to choose them as their partner in the future ultimatum game. Upon completing the partner choice task, participants watched the recorded screens of separate responders again and rated their perceived personality traits outside the scanner. Participants revisited the laboratory and replied to the surveys after completing the behavioural tasks at least two days apart but within two weeks. In addition, participants were asked whether they believed the recorded screens were from real responders and whether they correctly understood the structure of these tasks. All participants believed that the video clips were real but we excluded three participants because they misunderstood the rules or the structures of the task. All participants were debriefed and paid within three weeks of participation.

*1.2. The pre-task ultimatum game*

In the pre-task ultimatum game, a proposer determines the division of KRW 10,000 (USD 10) between the proposer and a responder in each trial. Both of them can earn the money of the amounts suggested by the proposer if the responder accepts the monetary offer from the proposer. On the other hand, neither of them can earn money if the responder rejects it. Participants performed ten practice trials of the ultimatum game where they took the part as the responders. Participants were told that all the monetary offers were suggested by prior participants who had participated as proposers. Unlike the typical ultimatum game, however, we simplified the monetary offer using only two ratios, 8:2 and 5:5, representing unfair and fair offers, respectively. Out of the 10 trials, 6 trials were unfair and 4 trials were fair. The participants were instructed that the result of one randomly chosen trial during this task would be given as their incentives. The detailed procedures of the pre-task ultimatum game are shown in **Supplementary Figure 1**.

This task was introduced for four reasons: 1) to estimate the individuals’ baseline tendency of tolerating unfairness by measuring the probability of accepting the unfair offers in the ultimatum game, 2) to make participants familiar with the ultimatum game, 3) to give them chances of taking the perspective of a responder, which might help them better understand the emotion or intention of the responder, and 4) to make it more believable that the video clips presented during the partner choice task are real. Nevertheless, we found no significant result associated with this individual baseline propensity of tolerating unfairness.

*1.3. The partner choice of the Ultimatum game*

The main task was designed to feature the decision time and decision type information from other decision-makers in the social decision-making. In each trial, participants first saw a video clip of ostensibly recorded screens from a previous responder of the ultimatum game, and then they were asked to decide whether they would choose the responder as their potential partner in the future ultimatum game. Participants were informed that their partners for the second ultimatum game would be randomly chosen from the responders whom they had chosen in this main task. The video clips of previous games contained a proposer’s offer amount (fair [5:5] or unfair [8:2] offer) followed by a responder’s decision (accept or reject). The duration between the two events was set at either 700ms (fast decision) or 3000ms (slow decision), which were determined by the results from an independent behavioural study.

To select optimal decision times that could be reliably perceived as fast or slow, we tested the perceived speed of a range of decision time from independent raters (N = 5). The independent raters watched the ostensibly recorded ultimatum game sequences where responders’ decision times varied across ten levels (i.e., 500, 700, 900, 1100, 1300, 1900, 2100, 2400, 2700, and 3000ms) after the onset of the offer from the proposer. Each level of decision time was presented twice for each type of response (i.e., rejecting an unfair offer, accepting an unfair offer, and accepting a fair offer). Participants’ ratings of perceived speed for each level were averaged across the same speed and the participants. Based on the distribution of these ratings across participants, we determined 700ms as the ‘slow’ speed because it significantly deviated from the mean (< lower 5%) and 3000ms as the ‘fast’ speed because it significantly deviated from the mean (> upper 5%). Besides, 700ms and 3000ms were respectively higher or lower than the upper or lower 10% of the distribution of our participants’ response time in the pre-task ultimatum game, which suggested that these speeds might feature the actual response time of fast or slow decision-makers.

The unfair conditions were further divided into four sub-conditions: fast accepter, fast rejecter, slow accepter, and slow rejecter conditions. The fair condition which accepting the 5:5 offer quickly was not the interest of our study, therefore, not included in the data analysis but presented to increase the believability of the cover story. Participants were asked to choose their partner for a proposer in half of the trials and a responder in the other half. Each of the 10 conditions included 15 trials, resulting in a total of 150 trials per participant.

Each trial of the task started with the initials of a responder (2s) who can be chosen as a partner. Next, a video clip was displayed showing the OFFER screen with a monetary offer from a proposer suggested to the responder and the decision options of ‘Accept’ and ‘Reject’. The OFFER screen was then followed by the OUTCOME screen where a chosen option was turned red after 700ms or 3000ms, depending on condition. The OUTCOME screen lasted 500ms, followed by the fixation-cross screen which appeared for 1-4s to distinguish the video clip and the subsequent events of the participant’s own choice (CHOICE screen). To emphasize that the video clips were from the recorded screens of the previous responders, the video screen looked identical to the monetary offer screen that participants had experienced in the pre-task ultimatum game. Therefore, the amount for the responder was displayed next to ‘Me’, not ‘Responder’. Instead, the OFFER and OUTCOME screens were outlined with thick black lines and a small red triangle that looked like a ‘Play’ sign and the word ‘Playing…’ was displayed on the left top corner of the screen so that participants easily distinguish the partner choice task from the video clip. On the CHOICE screen, the question ‘When the XXX (responder’s ID) is a ‘proposer’ (or ‘responder’), would you like to join the game later?’ with the option ‘YES’ and ‘NO’ displayed below. To make a choice, participants used a four-button MR-compatible response grip to press the first button with their index fingers or the second button with their middle fingers. When they pressed the key (or button), their chosen option turned red for 500ms, and the fixation screen with a crosshair was shown for 1-4s.

*1.4. The trait rating task*

We assessed the participants’ inferences on the personal traits of the responders depending on the decision types and the decision times of the responders during the ultimatum game. For this purpose, all participants performed the trait rating task where they watched the same video clips used in the partner choice task and rated the impression of the responders with regard to three social traits of warmth, competence, and likability using a four-point Likert scale.

The warmth and the competence were included because these are widely known to be the two fundamental dimensions of social perception for the social entity including the individuals and the social group (Cuddy et al., 2008; Fiske et al., 2002, 2007). Before the trait rating task, participants were informed of the definition of warmth and competence using the Korean-translated version of the word list from Fiske, Cuddy, & Glick (2002) and instructed to judge the responders for each of the traits with the definitions in mind. Besides, we asked participants to evaluate the responders based on the ‘likability’ to assess the bias in their preference aside from warmth and competence.

Upon completing the partner choice task, participants read the instruction and performed the trait rating task out of the scanner. In this task, participants watched additional recorded screens which were not included in the partner choice task. The trait rating task is analogous to the partner choice task except that the partner choice is replaced with partner evaluation. Therefore, instead of the CHOICE screen, it displayed the phrase of ‘In my opinion, XXX (responder ID) is’ and one of three traits, ‘warm’, ‘competent’ or ‘likeable,’ along with the four-point Likert scale (1: ‘not at all’ to 4: ‘totally agree’) below. Participants were asked to report their agreement with the complete sentence using the Likert scale, indicating the responder’s likelihood of having the suggested trait. The three traits were shown successively in a random order for each video clip. Participants were requested to decide within five seconds. The trait rating task consisted of eight trials and only four types of the responder who received unfair offers were suggested. Each type of responder was presented twice.

*1.5. Surveys and debriefing*

Within two weeks but at least two days apart from the behavioural task, participants revisited the laboratory and answered the surveys. The surveys included: (i) the questions asking current mood and arousal with the 7-point Likert scales, (ii) the Korean-translated 60-item version of HEXACO (Ashton & Lee, 2009), (iii) self-ratings of the participant’s traits in terms of every personality words from warmth and competence word list (Cuddy et al., 2008; Fiske et al., 2007; Lee et al., 2014) (iv) the sliding version of social value orientation test (Murphy et al., 2011).

In addition, participants were asked whether they believed the recorded screens were from real responders and guessed the hypotheses of this study. All participants believed that the video clips were real and were unable to exactly guess the hypotheses. Participants also had to describe the tasks or the structures of the experiments, but we excluded three participants because they misunderstood the rules or the structures of the task.

Participants were debriefed after answering the surveys and all of them were permitted to use their experimental data even after they knew the true goal of this study.

**Supplementary text 2. Analyses**

*2.1. Behavioural analyses*

To examine the influences of decision type, decision time, and the role of partner on the partner choice task, we executed a 2 (role of partner: proposer or responder) × 2 (decision type: accept or reject) × 2 (decision time: slow or fast) three-way repeated-measure ANOVA (rmANOVA) on the mean percentage of partner choice. Moreover, we also checked the effect of the type and the time of the responder within each role of the partner using a 2 (decision type: accept or reject) × 2 (decision time: slow or fast) two-way rmANOVA on the separated datasets for each partner role. We excluded the fair offer condition from the behavioural analyses because it was not of our interest and fundamentally different from the unfair offer conditions. The same analyses were conducted on the RT data. The mean RT of each condition was standardised across the conditions of partner’s role separately for each participant.

For the trait rating task, we hypothesized that the decision type and time would impact the perception of the social traits. Thus, a 2 (decision type: accept or reject) × 2 (decision time: slow or fast) two-way rmANOVA was implemented on the warmth, competence, and likability ratings, separately.

We also investigated whether the influence of warmth and competence rating would be different in each context of the partner’s role. Thus, we employed a generalized linear mixed model (GLMM) on the binary responses of the partner choices (0: No, 1: Yes), using the *‘glmer’* function of *‘lme4* (version 1.1-23)’ package (Bates et al., 2014) in R studio (version 1.2.5033). The fixed-effect predictors were the partner’s role (1: proposer, 2: responder) as a categorical variable, and the rating scores of perceived warmth and competence of each responder as the numerical variables, and their interactions. Also, we included the random effects of each participant accounting for the effect of the intercept, the warmth and the competence score, and the interaction of warmth and competence score to control the individual variances caused by the bias in general response tendency other than the changes according to the conditions. The perceived rating scores of warmth and competence were mean-centred such that the range of the scores changed from 1–4 to -1.5–1.5. The fixed and random effects of the GLMM model are listed in **Supplementary Table 2.**

We ran additional exploratory data analyses to examine the perceived ambiguity of each responder’s impression (See **Supplementary Text 3** for more details) and the temporal effects of decision time on the partner choice (see **Supplementary Text 4** ad **Supplementary Table 1**).

*2.2. fMRI data processing and analyses*

The fMRI data were collected by a 3T Siemens Magnetom Trio with a 12-channel head matrix coil for T2* and T1 images and a 32-channel head matrix coil for DTI images which was located in the Korea University Brain Imaging Center. T2*-weighted functional images were acquired using gradient-echo echo-planar pulse sequences (TR = 1900ms, TE = 30ms, FA = 90°; FOV = 240 mm; 80 × 80 matrix; 36 slices; voxel size = 3.0 × 3.0 × 3.0 mm^3^). We obtained the T1-weighted structural images (TR = 1900 ms; TE = 2.52 ms; FA = 9°; 256 × 256 matrix; voxel size = 1 × 1 × 1 mm^3^) and diffusion tensor image (TR = 3000 ms; TE = 70.0 ms; FA = 90°; 112 × 112 matrix; voxel size = 2 × 2 × 2 mm^3^) before or after the acquisition of functional images and the order of the structural image and DTI image acquisition was counterbalanced by the subject because of the different use of coils. The stimuli were presented through an MR-compatible LCD monitor mounted on the head coil(refresh rate: 85Hz; display resolution: 800 x 600 pixels; viewing angle: 30° horizontal, 23° vertical). The functional images were collected in a run which was lasted approximately 30 min.

All fMRI data were preprocessed and analyzed using the software of SPM12 (Wellcome Department of Imaging Neuroscience, London, United Kingdom). The functional images were corrected for the timing of slice acquisition and head motion by the realignment to the first volume. The images were normalized to the standard Montreal Neurological Institute (MNI) EPI template and resampled to 2 × 2 × 2mm3 and spatially smoothed using a Gaussian kernel with 8-mm full-with-half-maximum (FWHM).

To estimate the changes in the neural responses varied by the different events and the different responder conditions, we applied a general linear model (GLM) incorporating the onsets of the event when a responder received the monetary offer (OFFER onset), and the onsets of the event when the decision of a responder revealed (OUTCOME onset), and the onsets of the event when the participant had to chose the responder as a partner or not (CHOICE onset). In the OFFER onset, the onset of unfair offers and fair offers were included as separate regressors. We divided the OUTCOME onset into five regressors for fast rejecter, slow rejecter, fast accepter, slow accepter, fair offer receiver. The choice onset comprised ten regressors which also isolated the partner’s role (proposer or responder) for each of the five types of responders. The neuronal activations of the OFFER onset were fitted using a canonical hemodynamic response function (HRF) convolved with a boxcar function for the duration of the OFFER screen depending on the fast or slow condition (3000ms or 700ms), while the neuronal activations of the OUTCOME or CHOICE onsets were defined as the canonical HRF convolved with a stick function on the onset time.

Considering the behavioural results, we focused on the neural correlates related to two interaction effects: 1) The interaction between decision type and decision time when a participant observes the outcome of the responder’s decision, 2) The interaction between the partner’s role and the decision type when a participant chooses a potential partner as a proposer or a responder. First, to identify the neural area involving the decision type × decision time interaction effect, we created the participants’ first-level contrast maps of ‘[SlowRejecter ‒ FastRejecter] ‒ [SlowAccepter ‒ FastAccepter]’ on the OUTCOME onset and conducted the second-level one-sample T-test. Second, to identify the neural area related to the role × decision type interaction, we created the participants’ first-level contrast maps of ‘Proposer [Rejecter ‒ Accepter] – Responder [Rejecter – Accepter]’ on the CHOICE onset and conducted the second-level one-sample t-test. **Supplementary Table 3** reports the results of peak coordinates of the second-level one-sample T-test examining each main effect and the interaction effects related to the two interaction contrasts above at the p<0.001, uncorrected threshold.

Additionally, we investigated the main and the interaction effects by using second-level flexible-factorial ANOVA. First, the four first-level contrast maps (i.e., type [rejecter or accepter] × time [fast or slow]) from each subject estimated at the outcome onset were included in a second-level flexible-factorial ANOVA to investigate the main effects and the interaction effect of the type and time factors. Second, another second-level flexible-factorial ANOVA was applied on the eight first-level contrast maps (i.e., role [proposer or responder] × type [rejecter or accepter] × time [fast or slow]) from each subject estimated at the choice onset to investigate the main effects and the interaction effect of the role and type factors. Consistent with the original one-sample t-test, the aMCC and the RMPFC, which had shown significant two-way interaction effects (i.e., type × time interaction effect at the OUTCOME onset, role × type interaction effect at the CHOICE onset), showed similar patterns of theinteraction effect, although neither of them survived the correction for multiple comparisons. Specifically, the aMCC (x = -10, y = 28, z = 26, cluster size = 145) showed a type × time interaction effect, P_cluster-FWE_ = 0.072 and the RMPFC (x = 0, y = 54, z = 0, cluster size = 151) showed a role × type interaction effect, F = 21.14 P_peak-FWE_ = 0.122. The statistics and peak coordinates of the clusters from two ANOVAs were reported in **Supplementary Table 4** at *p* < 0.001, uncorrected.

In addition to our main analyses, we explored the neural activations which might reflect the individual differences in the subjective warmth perceptions between the rejecters and the accepters. Considering the higher warmth ratings and partner choice rates for the accepters compared to the rejecters, the process of partner choice might involve neural activities which might be less influenced by the context or role. We focused on the subregions of mPFC that are highly related to value computation and social decision-making (Bartra et al., 2013; Kable & Glimcher, 2007; Kim, 2020; Kim et al., 2007). The subjective differences in warmth perception were calculated by subtracting the warmth ratings of the accepters from those of the rejecters (i.e., ‘rejecter warmth – accepter warmth’). The warmth ratings of the four responders were normalized within each participant. This score regressed the contrast map of ‘Rejecter – Accepter’ of the OUTCOME and CHOICE onset. The resulting maps of these multiple regressions were thresholded the small volume correction using the binary mask map of mPFC which defined the subregions from the functional coactivation map (De La Vega et al., 2016).In the behavioural and fMRI data analyses, we excluded the trials in which the participants missed any part of the entire video clip to ensure that full information about the responders was available for impression formation. Since the interest of our study is the changes of the neural pattern after a person observed the decision choice and decision time of other people, the participant’s choice is only meaningful when he or she watched the whole decision process of the other responder indeed. Thus, we had to exclude the trials where people could not watch the video clip stimuli but they chose an option anyhow. The trials to be excluded were defined based on the two standards: (i) the trial in which a participant could not see the offer screen more than the 2000ms from the start of OFFER onset because he or she might be unable to tell the difference of the decision time, (ii) the trial in which a participant could not see the OUTCOME screen because he or she cannot infer what kind of a person the responder would be. The independent experimenter who had not known the research hypothesis watched the eye tracker on-line outside of the scanner for the whole session of a functional run and recorded the time point to be excluded. The number of participants whose trials had been excluded for more than 10% of their whole 150 trials was seven. One participant was excluded because the participant barely watched the screen from the start trial.

The neuroimaging results reported in this study were corrected in the threshold of p < 0.05 for the peak-level family-wise error rate (FWE) or the cluster-level FWE which were defined in the initial uncorrected p < 0.001.

**Supplementary References**

Ashton, M. C., & Lee, K. (2009). The HEXACO-60: A short measure of the major dimensions of personality. *Journal of Personality Assessment*, *91*(4), 340–345. https://doi.org/10.1080/00223890902935878

Bartra, O., McGuire, J. T., & Kable, J. W. (2013). The valuation system: A coordinate-based meta-analysis of BOLD fMRI experiments examining neural correlates of subjective value. *NeuroImage*, *76*, 412–427. https://doi.org/10.1016/J.NEUROIMAGE.2013.02.063

Bates, D., Mächler, M., Bolker, B., & Walker, S. (2014). Fitting Linear Mixed-Effects Models using lme4. *Journal of Statistical Software*, *67*(1). https://arxiv.org/abs/1406.5823v1

Cuddy, A. J. C., Fiske, S. T., & Glick, P. (2008). Warmth and Competence as Universal Dimensions of Social Perception: The Stereotype Content Model and the BIAS Map. In *Advances in Experimental Social Psychology* (Vol. 40). Academic Press. https://doi.org/10.1016/S0065-2601(07)00002-0

De La Vega, A., Chang, L. J., Banich, M. T., Wager, T. D., & Yarkoni, T. (2016). Large-scale meta-analysis of human medial frontal cortex reveals tripartite functional organization. *Journal of Neuroscience*, *36*(24), 6553–6562. https://doi.org/10.1523/JNEUROSCI.4402-15.2016

Fiske, S. T., Cuddy, A. J. C., & Glick, P. (2007). Universal dimensions of social cognition: warmth and competence. *Trends in Cognitive Sciences*, *11*(2), 77–83. https://doi.org/10.1016/J.TICS.2006.11.005

Fiske, S. T., Cuddy, A. J. C., Glick, P., & Xu, J. (2002). A model of (often mixed) stereotype content: Competence and warmth respectively follow from perceived status and competition. *Journal of Personality and Social Psychology*, *82*(6), 878–902. https://doi.org/10.1037/0022-3514.82.6.878

Kable, J. W., & Glimcher, P. W. (2007). The neural correlates of subjective value during intertemporal choice. *Nature Neuroscience*, *10*(12), 1625–1633. https://doi.org/10.1038/nn2007

Kim, H. (2020). Stability or Plasticity? – A Hierarchical Allostatic Regulation Model of Medial Prefrontal Cortex Function for Social Valuation. *Frontiers in Neuroscience*, *14*(March), 31. https://doi.org/10.3389/fnins.2020.00281

Kim, H., Adolphs, R., O’Doherty, J. P., & Shimojo, S. (2007). Temporal isolation of neural processes underlying face preference decisions. *Proceedings of the National Academy of Sciences*, *104*(46), 18253–18258. https://doi.org/10.1073/PNAS.0703101104

Lee, M. W., Sul, S. H., & Kim, H. J. (2014). *The Impact of Moral Decision Style on Impression Formation Minwoo*.

Murphy, R. O., Ackermann, K. A., & Handgraaf, M. J. J. (2011). Measuring Social Value Orientation. *Judgment and Decision Making*, *6*(8), 771–781. https://doi.org/10.2139/ssrn.1804189

**Supplementary Text 3. The ambiguity of each responder’s value**

The fMRI results showed the distinguished activation of aMCC after watching the slow rejecter compared to other responders. Also, the differences in trait perception between slow and fast rejecters are greater than those between slow and fast accepters, though the trait ratings in respect to the rejecters were less preferable to the accepters. Therefore, it might be possible to infer that the high activation of aMCC in the slow rejecter condition might be accounted for by the conflicting value of slow rejecter, considering that the engagement of DMPFC, especially aMCC, might be related to the enhanced conflict or ambiguity evoked from the value competition.

For that reason, the additional behavioural data analyses were executed to try to figure out whether the slow rejecter could entail a more ambiguous or conflicted value in contrast to the other types of responders. For example, the value of the slow rejecter could be comparable to the mid-point, the decision threshold, of ratings or decision-making, which might elicit more conflicts.

First, the conflicting value of the slow rejecter could be derived from the perceived traits. We checked if the perceived trait ratings of each responder might be different from the midpoint value (2.5 from the four-point Likert Scale). Since the decision type × decision time interaction effect on aMCC and the corresponding interaction effect on likability rating was correlated, we compared the likability of each condition with 2.5 using an One-sample T-Test. As the results, only the mean likability ratings of slow rejecters were not statistically different from 2.5 (Slow Rejecter: *t*(39) = -1.62, p=0.114; Fast Rejecter: *t*(39)=-4.03, p<0.001; Slow Accepter: *t*(39)=2.63, *p*=0.012; Fast Accepter: *t*(39)=2.19, *p*=0.035). This might suggest the relatively ambiguous value of slow rejecter. However, the warmth rating which also showed the significant decision type × decision time interaction did not show these patterns (Slow Rejecter: *t*(39) = -4.07, p=0.000; Fast Rejecter: *t*(39)=-11.90, *p*<0.001; Slow Accepter: *t*(39)=1.534, *p*=0.133; Fast Accepter: *t*(39)=3.955, *p*<0.001).

Second, the value of the slow rejecter could be inferred from the average willingness to choose a partner. The average rates of partner choice across the roles might represent the value of each responder. We conducted a One-sample T-Test comparing the percentage of each responder being chosen as a partner with 50% which was the threshold of partner preference. Similar to the results of trait rating, the slower rejecter was obviously not different from the midpoint (*t*(38)=-0.40, *p*=0.690), even though the partner choice rate of the fast rejecter was slightly not different to the 50% (Fast Rejecter: *t*(38)=-1.92, *p*=0.062; Slow Accepter: *t*(38)=5.26, *p*<0.001; Fast Accepter: *t*(38)=5.04, *p*<0.001).

From these results, it might be possible that the value of slow rejecter might be more ambiguous to the participants, and this might be represented as the aMCC activation pattern.

**Supplementary Text 4. No temporal change of the influence of decision time on the partner choice**

In our study, unlike the decision type, the influence of the decision time on the partner-choice was not significant, which could be due to the differences between them in the degree of informational value. However, there is another possibility that participants have learned to ignore the decision time information as they had experienced over the course of the task that the potential partners’ decision time was ultimately orthogonal to their decisions and thus non-predictive (e.g., slow and fast partners accepted and rejected at the same rates). If this hypothesis was correct, the effect of decision time on partner choice would be significant at least at the early period of the task but decrease over time. To test this hypothesis, we investigated whether the decision time effect or its interaction with decision type changed across the early-, mid- and late parts of the experiment, by dividing the experimental trials into three blocks of 50 trials (i.e., 2 roles × 5 responders × 5 repetitions). In this process, one participant was additionally excluded due to the omission of choice data in some of the conditions in block 3. Then, the average rates of partner choice in each unfair condition were analyzed by the 2 (role: proposer or responder) × 2 (type: rejecter or accepter) × 2 (time: fast or slow) repeated-measure ANCOVA with the covariate of block numbers (1, 2, and 3). However, the block did not influence any effects of the decision time or its interaction effects (time × block interaction effect: F(1,114) = 0.65, p = 0.423; role × time × block interaction effect: F(1,114) = 3.391, p = 0.068; type × time × block interaction effect: F(1,114) = 0.646, p = 0.423; role × type × time × block interaction effect: F(1,114) = 0.098, p = 0.755) (**Supplementary Table 1**).

Additionally, we checked the 2 (type: rejecter or accepter) × 2 (time: fast or slow) repeated-measure ANOVA on each role of each block. But the time main effect or the type × time interaction effect of each block was also not statistically significant. Rather, the time main effect on the ‘proposer’ choice seemed to increase, because it was statistically significant in block 3, but not in block 1 or 2 (block 1: F(1,38) = 0.004, p = 0.949; block 2: F(1,38) = 1.767, p = 0.192; block 3: F(1,37) = 4.491, p = 0.041). The time main effect on the responder choice was not significant in any of the three blocks (**Supplementary Table 1** and **Supplementary Figure 2**). This might imply that decision time is less salient than decision type therefore is less intuitively considered for partner choice at least in the early period of the task. However, participants may have paid more and more attention to this information as they had gained abundant evidence for the distribution of their potential partner pool, especially in the condition where the decision type information does not accurately predict the behaviour of potential partners in the future ultimatum game (i.e., proposer choice condition).

Based on these results, we believe it unlikely that the null effect of decision time on the partner choice was due to the participants’ learning that the decision time is not informative.

**Supplementary Figure 1. Task screens of the pre-task ultimatum game**


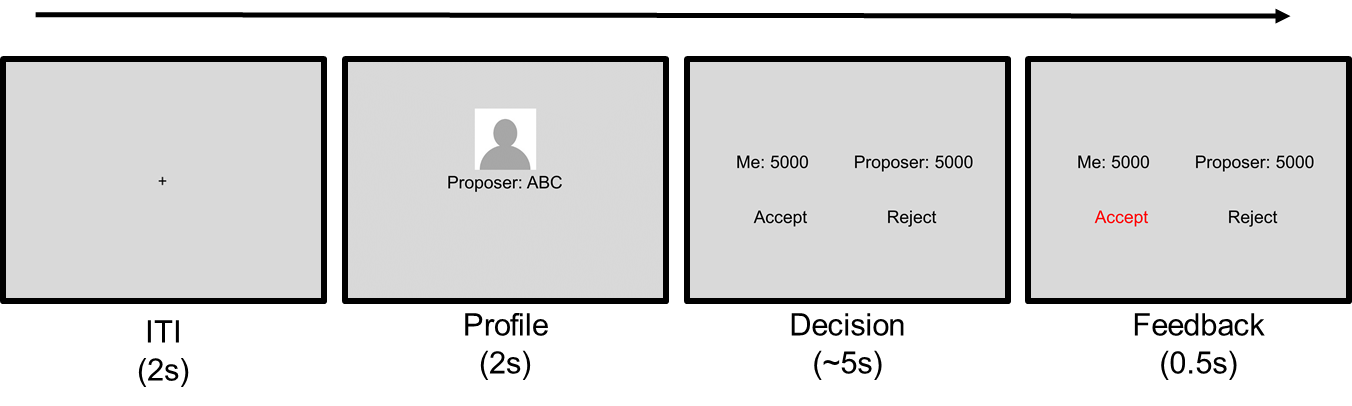


Each trial started with the proposer’s ID presented on the screen for 2s, which was then followed by the proposer’s offer amount, and the options of ‘Accept’ and ‘Reject’ appeared together. The amount for the participant was presented next to ‘Me’ (e.g. Me: 2000) on the upper left and the amount for the proposer was presented next to ‘Proposer’ (e.g. Proposer: 8000) on the upper right. Participants were prompted to decide whether they accept or reject the offer within 5s by pressing ‘F’ or ‘J’ on a keyboard. Then the chosen option turned red displayed for 500ms.

**Supplementary Figure 2. The graphs showing changes in the partner-choice rate across blocks.**

**.**


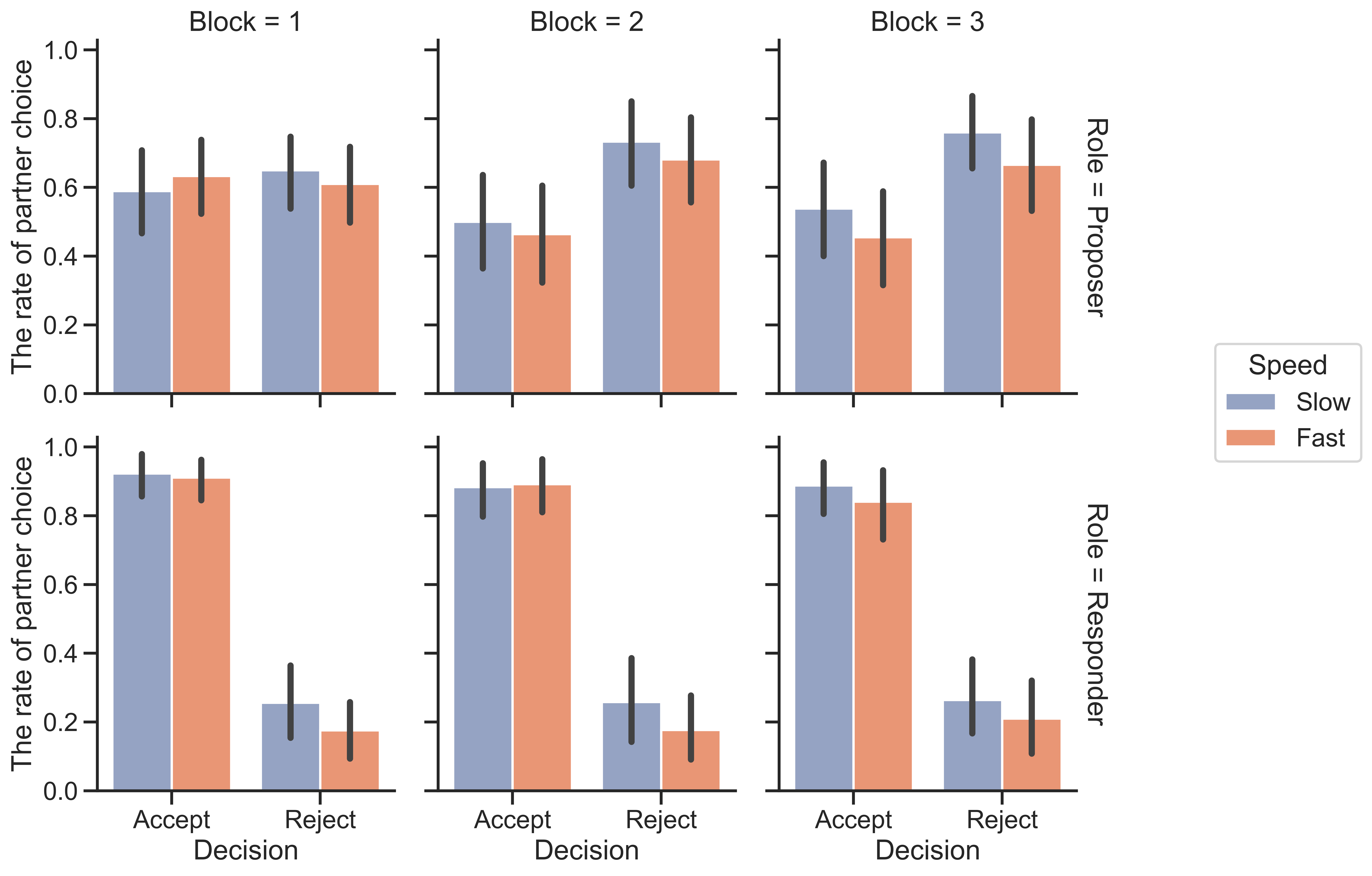


The rate of choosing a partner as a proposer or responder in each block. The row of the graph indicates the role of partner (upper: proposer, lower: responder) and the column of the graph indicates the block number (left: block 1, middle: block 2, right: block 3). The blocks were split by the early-, mid- and late parts of the task, which consist of 50 trials each (i.e., 2 roles × 5 responders × 5 repetitions). We investigated whether the decision time effect or its interaction with decision type had decreased over time (i.e., block) because participants had learned that the decision time information was not predictive where the structure of the task had orthogonalized the decision type and time. However, the block did not influence any effects of the decision time or its interaction effects. The error bars indicate the 95% confidence interval.

**Supplementary Figure 3. F-contrast statistical maps from the flexible-factorial ANOVAs**

**
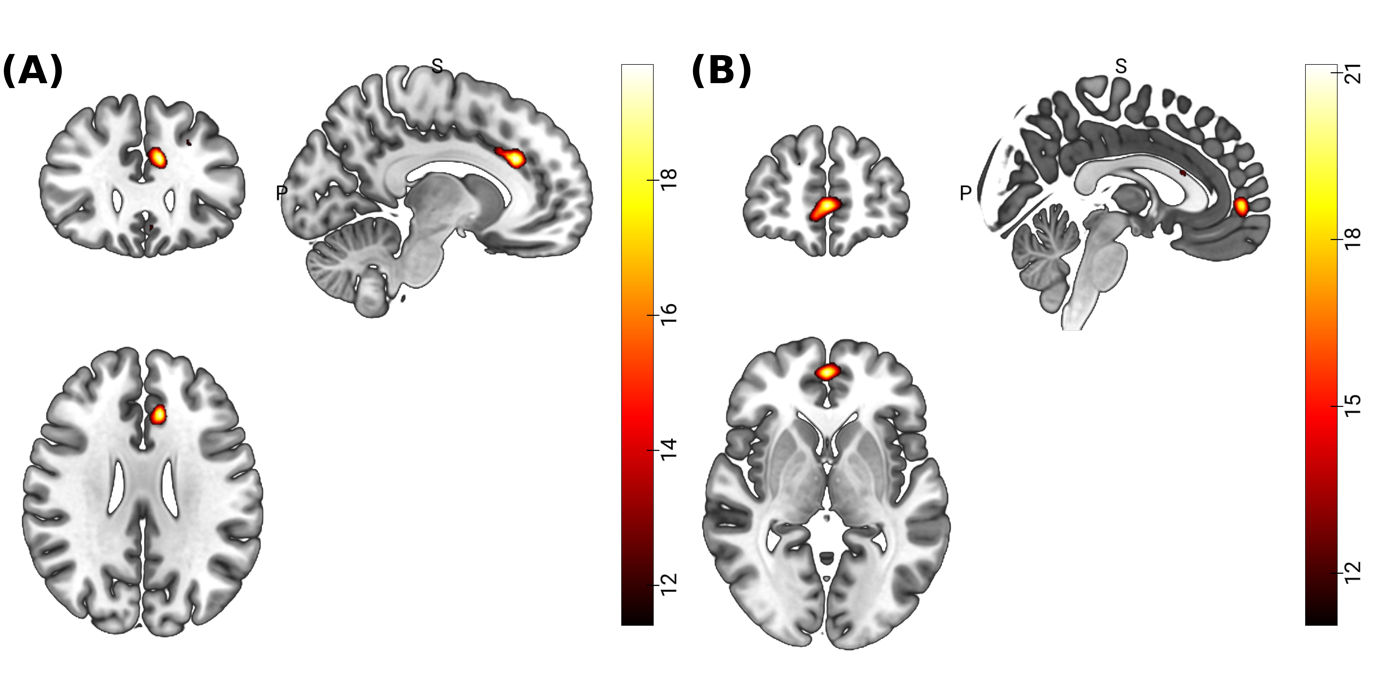
**

The result maps from the F-contrast of the flexible-factorial ANOVA. (uncorrected, P<0.001) (A). The type × time interaction map on the OUTCOME onset. (B). The role × type interaction map on the CHOICE onset. Both analyses could not show the statistically significant results because the effect sizes of aMCC(A) and rmPFC(B) decreased compared to those of one-sample T-test.

**Supplementary Figure 4. Role × type interaction effect on CHOICE onset after controlling the RT**


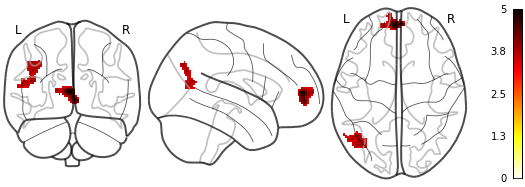


The contrast map of Proposer [Rejecter – Accpeter] – Responder [Rejecter – Accepter] on CHOICE onset after controlling for the RT, FWE-corrected at p < 0.05 for cluster-level inference. Two clusters showed the significant activations: RMPFC (*x* = 0, *y* = 52, *z* = 0; P_cluster-FWE corrected_ = 0.010, voxel size = 283), Left middle temporal gyrus (*x* = -38, *y* = -66, *z* = 12; P_cluster-FWE corrected_ = 0.025, voxel size = 228).

**Supplementary Table 1. The results from the repeated-measure ANCOVA for testing the temporal (i.e., Block) effect.**

| **Effect** | **DF** | **DFe** | **F-value** | **P-value** | **Partial η2** |
| --- | --- | --- | --- | --- | --- |
| **2(Role) × 2(Type) × 2(Time) × 2(Block) repeated-measure ANCOVA** | | | | | |
| Role | 1 | 114 | 1.03 | 0.314 | 0.01 |
| Role × block | 1 | 114 | 0.01 | 0.927 | 0.00 |
| Type | 1 | 114 | 13.54 | 0.000 | 0.11 |
| Type × block | 1 | 114 | 1.93 | 0.167 | 0.02 |
| Time | 1 | 114 | 0.03 | 0.876 | 0.00 |
| Time × block | 1 | 114 | 0.65 | 0.423 | 0.01 |
| Role × Type | 1 | 114 | 15.99 | 0.000 | 0.12 |
| Role × Type × block | 1 | 114 | 0.76 | 0.385 | 0.01 |
| Role × Time | 1 | 114 | 3.26 | 0.074 | 0.03 |
| Role × Time × block | 1 | 114 | 3.39 | 0.068 | 0.03 |
| Type × Time | 1 | 114 | 2.17 | 0.144 | 0.02 |
| Type × Time × block | 1 | 114 | 0.65 | 0.423 | 0.01 |
| Role × Type × Time | 1 | 114 | 0.00 | 0.976 | 0.00 |
| Role × Type × Time × block | 1 | 114 | 0.10 | 0.755 | 0.00 |
| **2(Type) × 2(Time) repeated-measure ANOVA for each block** | | | | | |
| **Block1** | | | | | |
| ***Proposer*** |  |  |  |  |  |
| Type | 1 | 38 | 0.04 | 0.846 | 0.00 |
| Time | 1 | 38 | 0.00 | 0.949 | 0.00 |
| Type × Time | 1 | 38 | 2.45 | 0.126 | 0.06 |
| ***Responder*** |  |  |  |  |  |
| Type | 1 | 38 | 135.07 | 0.000 | 0.78 |
| Time | 1 | 38 | 1.69 | 0.201 | 0.04 |
| Type × Time | 1 | 38 | 2.07 | 0.159 | 0.05 |
| **Block2** | | | | | |
| ***Proposer*** |  |  |  |  |  |
| Type | 1 | 38 | 4.11 | 0.050 | 0.10 |
| Time | 1 | 38 | 1.77 | 0.192 | 0.04 |
| Type × Time | 1 | 38 | 0.07 | 0.788 | 0.00 |
| ***Responder*** |  |  |  |  |  |
| Type | 1 | 38 | 73.80 | 0.000 | 0.66 |
| Time | 1 | 38 | 1.60 | 0.213 | 0.04 |
| Type × Time | 1 | 38 | 2.36 | 0.132 | 0.06 |
| **Block3** | | | | | |
| ***Proposer*** |  |  |  |  |  |
| Type | 1 | 37 | 4.42 | 0.042 | 0.11 |
| Time | 1 | 37 | 4.49 | 0.041 | 0.11 |
| Type × Time | 1 | 37 | 0.09 | 0.761 | 0.00 |
| ***Responder*** |  |  |  |  |  |
| Type | 1 | 37 | 64.37 | 0.000 | 0.64 |
| Time | 1 | 37 | 1.68 | 0.204 | 0.04 |
| Type × Time | 1 | 37 | 0.30 | 0.589 | 0.01 |

**Supplementary Table 2. GLMM results of fixed and random effects**

| Fixed-effects | **Effect** | **b(SE)** |
| --- | --- | --- |
|  | **Intercept** | **0.75(0.23) **** |
|  | **Role** | **0.21(0.08) *** |
|  | Warmth | 0.30(0.31) |
|  | Competence | 0.01(0.27) |
|  | **Role × Warmth** | **1.60(0.10) ***** |
|  | **Role × Competence** | **-0.58(0.09) ***** |
|  | Warmth × Competence | -0.05(0.13) |
|  | Role × Warmth × Competence | 0.17(0.10) |
| Random-effects | **Effect** | **Variance(SD)** |
|  | Intercept | 1.67(1.29) |
|  | Warmth | 2.89(1.70) |
|  | Competence | 2.00(1.42) |
| Model Statistics | AIC | 4480.2 |
|  | BIC | 4570 |
|  | Loglikelihood | -2226.1 |

* Model description: Partner Choice (1:Yes, 0: No) ~ Role(1:Proposer, 2:Responder) × Warmth (Mid-point (2.5) centered) × Competence (Mid-point centered) + (1+Warmth*Competence | subject).

* AIC = Akaike Information Criterion, BIC = Baesian Information Criterion. Significance: ‘***’, p < 0.001; ‘**’, p < 0.01; ‘*’, p < 0.05, ‘.’, p < 0.1.

**Supplementary Table 3. The peak coordinates of the second-level one-sample t-test**

| **Anatomical description** | **Cluster-level P_FWE_** | **K** | **T** | **x** | **y** | **z** |
| --- | --- | --- | --- | --- | --- | --- |
| **Outcome onset: Rejecter - Accepter** | | | | | | |
| **Cerebellum, R** | **0.024*** | 204 | 4.38 | 26 | -46 | -20 |
|  |  |  | 4.09 | 16 | -48 | -26 |
|  |  |  | 3.88 | 12 | -50 | -18 |
| Superior/mid temporal gyrus, R | 0.705 | 42 | 4.12 | 66 | -36 | 18 |
|  | 0.963 | 16 | 4.06 | 64 | -44 | 2 |
| Pallidum, R | 0.866 | 28 | 4.01 | 12 | 2 | -2 |
| Cerebellum, L | 0.937 | 20 | 3.85 | -8 | -68 | -10 |
| Middle cingulate Gyrus, L | 0.988 | 10 | 3.78 | -8 | 16 | 32 |
| **Outcome onset: Accepter - Rejecter** | | | | | | |
| Insula, L | 0.988 | 10 | 3.81 | -38 | 12 | -12 |
| **Outcome onset: Slow - Fast** | | | | | | |
| **Superior/middle frontal gyrus, R** | **0.000*** | 4492 | **7.8*** | 22 | 42 | 38 |
|  |  |  | **7.34*** | 32 | 34 | 42 |
|  |  |  | **7.14*** | 62 | -4 | 0 |
| **Superior/middle frontal gyrus, L** | **0.000*** | 2137 | **7.52*** | -42 | 30 | -18 |
|  |  |  | **5.89*** | -24 | 56 | 26 |
|  |  |  | 5.49 | -34 | 58 | 6 |
| **Superior temporal gyrus, L** | **0.000*** | 487 | **7.23*** | -62 | -14 | 10 |
|  |  |  | 5.19 | -60 | -2 | 2 |
|  |  |  | 4.93 | -60 | -28 | 14 |
|  | 0.254 | 85 | 4.94 | -42 | -22 | 2 |
| **Middle/inferior temporal gyrus, R** | **0.000*** | 1233 | **6.75*** | 54 | -64 | 0 |
|  |  |  | **6.19*** | 58 | -58 | -8 |
|  |  |  | 5.17 | 58 | -24 | 50 |
| **Fusiform gyrus/ParaHippocampal gyrus, R** | **0.044*** | 159 | **5.99*** | 28 | -36 | -20 |
|  |  |  | 4.94 | 32 | -24 | -24 |
| **Angular gyrus/Middle occipital gyrus, L** | **0.000*** | 500 | **5.96*** | -44 | -70 | 34 |
|  |  |  | 5.47 | -48 | -76 | 18 |
|  |  |  | 5.27 | -54 | -60 | 38 |
| **Cerebellum, R** | **0.001*** | 361 | **5.75*** | 38 | -74 | -42 |
|  |  |  | 4.8 | 24 | -82 | -34 |
|  |  |  | 4.55 | 30 | -76 | -38 |
|  | 0.98 | 13 | 3.82 | 22 | -64 | -34 |
| **Cerebellum, L** | **0.000*** | 627 | **5.7*** | -36 | -78 | -40 |
|  |  |  | 5.46 | -24 | -72 | -38 |
|  |  |  | 5.21 | -26 | -80 | -40 |
|  | 0.882 | 26 | 4.45 | -32 | -48 | -42 |
|  | 0.953 | 18 | 3.92 | 0 | -80 | -26 |
|  | 0.774 | 35 | 3.84 | -10 | -62 | -16 |
|  |  |  | 3.75 | -18 | -62 | -20 |
|  |  |  | 3.37 | 2 | -66 | -18 |
|  | 0.799 | 33 | 3.7 | -26 | -50 | -30 |
| **Superior frontal gyrus, Medial, L (anterior mid-cingulate cortex)** | **0.000*** | 715 | 5.35 | 0 | 32 | 34 |
|  |  |  | 4.88 | 2 | 22 | 16 |
|  |  |  | 4.86 | 2 | 24 | 34 |
| **Putamen, L** | **0.000*** | 543 | 5.27 | -8 | -2 | 8 |
|  |  |  | 4.71 | -20 | -2 | 4 |
|  |  |  | 4.6 | -28 | -6 | 2 |
| Anterior orbital gyrus, R | 0.848 | 29 | 5.07 | 22 | 42 | -20 |
| Middle temporal gyrus, L | 0.748 | 37 | 5.05 | -62 | -34 | -16 |
|  | 0.555 | 52 | 4.36 | -62 | -12 | -20 |
| **Pregenual anterior cingulate cortex, L** | **0.022*** | 189 | 4.7 | -6 | 44 | 16 |
|  |  |  | 4.09 | -6 | 48 | 4 |
|  |  |  | 3.39 | -2 | 52 | 20 |
| Vermis | 0.254 | 85 | 4.59 | 6 | -50 | 6 |
| **Precuneus, L** | **0.016*** | 205 | 4.54 | 0 | -64 | 40 |
|  |  |  | 3.42 | -10 | -50 | 30 |
| Calcarine/Middle occipital cortex, R | 0.617 | 47 | 4.04 | 22 | -96 | 4 |
|  |  |  | 3.6 | 28 | -86 | 0 |
| Middle cingulate cortex, L | 0.543 | 53 | 3.84 | -2 | -14 | 40 |
|  |  |  | 3.35 | 0 | -22 | 30 |
| Temporal pole: superior temporal gyrus, R | 0.99 | 10 | 3.74 | 32 | 20 | -32 |
| Superior frontal gyrus, L | 0.93 | 21 | 3.73 | -16 | 40 | 46 |
| Fusiform gyrus, R | 0.93 | 21 | 3.66 | 42 | -38 | -20 |
| Ventral striatum, R | 0.99 | 10 | 3.63 | 12 | 16 | -8 |
| Outcome onset: Fast - Slow | | | | | | |
| **Lingual gyrus, L** | **0.000*** | 2633 | 7.78* | -14 | -78 | -12 |
|  |  |  | 7.17* | 12 | -76 | 4 |
|  |  |  | 6.93* | -10 | -78 | -4 |
| **Middle occipital gyrus, L** | **0.003*** | 292 | 4.89* | -32 | -88 | 16 |
|  |  |  | 4.75* | -28 | -78 | 18 |
|  | 0.474 | 59 | 4.46* | -28 | -64 | 32 |
| Precentral gyrus, R | 0.403 | 66 | 4.84 | 42 | 4 | 32 |
| Middle frontal gyrus, L | 0.267 | 83 | 4.56 | 36 | -2 | 52 |
| Middle occipital gyrus, R | 0.189 | 97 | 4.51 | 30 | -86 | 18 |
|  |  |  | 4.26 | 24 | -90 | 24 |
| **Precentral gyrus, L** | **0.044*** | 159 | 4.43 | -38 | 0 | 30 |
|  |  |  | 4.39 | -38 | -4 | 46 |
| Inferior parietal gyrus, L | 0.912 | 23 | 3.86 | -26 | -52 | 44 |
| Insula, L | 0.953 | 18 | 3.84 | -32 | 22 | 6 |
|  | 0.98 | 13 | 3.75 | -36 | 20 | -2 |
| **Outcome onset: (Rejecter - Accepter) * (Slow - Fast)** | | | | | | |
| **Anterior cingulate cortex, supracallosal, L (anterior mid-cingulate cortex)** | **0.028*** | 208 | 5.12 | -10 | 30 | 28 |
|  |  |  | 4.17 | -10 | 20 | 34 |
| Middle temporal gyrus, L | 0.438 | 69 | 4.82 | -48 | -12 | -14 |
| Fusiform gyrus, R | 0.838 | 31 | 4.24 | 22 | -48 | -14 |
| Middle frontal gyrus, R | 0.904 | 24 | 4.02 | 32 | 44 | 18 |
| Temporal pole: superior temporal gyrus, R | 0.949 | 18 | 4 | 48 | 8 | -18 |
| Middle cingulate cortex, R | 0.542 | 58 | 3.98 | 4 | 10 | 36 |
|  |  |  | 3.6 | 4 | 20 | 30 |
| Insula, R | 0.921 | 22 | 3.94 | 40 | 10 | -8 |
| Vermis | 0.943 | 19 | 3.91 | 6 | -52 | -6 |
| Superior frontal gyrus (dorsolateral prefrontal cortex), L | 0.967 | 15 | 3.83 | -18 | 52 | 12 |
| Cerebellum, L | 0.961 | 16 | 3.79 | -26 | -42 | -24 |
| Postcentral gyrus, L | 0.98 | 12 | 3.77 | -54 | -6 | 18 |
| **Outcome onset: (Rejecter - Accepter) * (Slow - Fast)** | | | | | | |
| no significant results |  |  |  |  |  |  |
| **Choice onset: Proposer - Responder** | | | | | | |
| no significant results |  |  |  |  |  |  |
| **Choice onset: Responder - Proposer** | | | | | | |
| **Precuneus, L / Middle cingulate cortex, R** | **0.000*** | 567 | **5.64*** | 0 | -48 | 46 |
|  |  |  | 4.91 | 10 | -36 | 40 |
|  |  |  | 4.82 | 4 | -36 | 32 |
| **Precentral gyrus, L** | **0.042*** | 191 | 5.32 | -58 | 2 | 20 |
|  |  |  | 4.74 | -50 | 0 | 36 |
| Inferior frontal gyrus, pars orbitalis, R | 0.887 | 26 | 5.04 | 50 | 22 | -8 |
| **Lingual gyrus, R/Calcarine cortex, L** | **0.002*** | 367 | 5.02 | 8 | -58 | 2 |
|  |  |  | 4.02 | -6 | -52 | 6 |
| Supplementary motor area/Superior frontal gyrus(dorsolateral prefrontal cortex), R | 0.068 | 165 | 4.86 | 14 | -10 | 52 |
|  |  |  | 3.88 | 20 | 2 | 60 |
|  |  |  | 3.72 | 12 | -4 | 58 |
| Middle occipital gyrus, L | 0.849 | 30 | 4.61 | -28 | -84 | 30 |
| **Middle cingulate gyrus** | **0.019*** | 235 | 4.51 | -2 | 4 | 34 |
|  |  |  | 4.12 | -4 | -6 | 38 |
|  |  |  | 3.93 | 6 | -8 | 36 |
| Superior frontal gyrus, dorsolateral, R | 0.204 | 109 | 4.48 | 26 | 44 | 42 |
|  |  |  | 4.31 | 34 | 46 | 28 |
|  |  |  | 3.51 | 26 | 34 | 38 |
| Inferior frontal gyrus, triangular part | 0.777 | 37 | 4.45 | 48 | 34 | 8 |
| Middle occipital gyrus, L | 0.859 | 29 | 4.41 | -34 | -88 | 8 |
| Inferior occipital gyrus, L | 0.281 | 93 | 4.33 | -44 | -74 | -8 |
| Superior frontal gyrus, medial orbital, L | 0.829 | 32 | 4.18 | -8 | 50 | -14 |
|  |  |  | 3.39 | 0 | 52 | -14 |
| Precuneus, L | 0.711 | 43 | 3.99 | -10 | -60 | 20 |
| Superior temporal gyrus, R | 0.96 | 16 | 3.9 | 52 | -24 | 0 |
| Cerebellum, R | 0.979 | 12 | 3.88 | 30 | -74 | -22 |
| Precuneus, R | 0.92 | 22 | 3.88 | 12 | -60 | 22 |
| Middle cingulate cortex, R | 0.92 | 22 | 3.8 | 8 | -16 | 36 |
| Middle frontal gyrus, L | 0.979 | 12 | 3.74 | -26 | 36 | 24 |
| Cerebellum, L | 0.96 | 16 | 3.72 | -14 | -42 | -14 |
|  | 0.904 | 24 | 3.68 | -4 | -68 | -10 |
| Choice onset: Rejecter - Accepter | | | | | | |
| Inferior temporal gyrus, R | 0.913 | 23 | 4.31 | 50 | -14 | -22 |
| Insula/Temporal pole, L | 0.091 | 144 | 4.3 | -32 | 20 | -8 |
|  |  |  | 3.95 | -30 | 20 | 0 |
|  |  |  | 3.66 | -44 | 20 | -14 |
| **Supplementary motor area, R/Superior frontal gyrus, medial(DMPFC)** | **0.027*** | 206 | 4.29 | 10 | 24 | 54 |
|  |  |  | 4.04 | 6 | 26 | 46 |
|  |  |  | 3.92 | -4 | 32 | 42 |
| Supplementary motor area, R | 0.725 | 41 | 4.06 | -2 | 20 | 60 |
| Middle frontal gyrus, L | 0.204 | 105 | 4.05 | 40 | 10 | 44 |
| Inferior parietal gyrus, R | 0.76 | 38 | 3.99 | 44 | -50 | 40 |
| Cerebellum, R | 0.936 | 20 | 3.91 | 26 | -70 | -28 |
|  | 0.904 | 24 | 3.84 | 10 | -74 | -28 |
| Superior frontal gyrus, L | 0.886 | 26 | 3.86 | -24 | 58 | -8 |
| Superior frontal gyrus, R | 0.886 | 26 | 3.81 | 18 | 44 | 34 |
| Angular gyrus, L | 0.714 | 42 | 3.81 | -42 | -56 | 28 |
| Calcarine cortex, L | 0.679 | 45 | 3.78 | -12 | -88 | 2 |
| Middle occipital gyrus, L |  |  | 3.42 | -16 | -94 | 6 |
| Inferior frontal gyrus, L | 0.921 | 22 | 3.7 | -46 | 16 | 0 |
| Calcarine cortex, R | 0.691 | 44 | 3.7 | 18 | -96 | 4 |
| Middle frontal cortex, R | 0.904 | 24 | 3.68 | 32 | 20 | 50 |
| **Choice onset: Accepter - Rejecter** | | | | | | |
| no significant results |  |  |  |  |  |  |
| **Choice onset: (Proposer - Responder) * (Rejecter - Accepter)** | | | | | | |
| **Superior frontal gyrus, medial (rostromedial prefrontal cortex), R** | 0.058 | 181 | **5.63*** | 0 | 54 | 0 |
| Superior frontal gyrus, R | 0.958 | 16 | 3.59 | 16 | 54 | 24 |
| **Choice onset: (Responder - Proposer) * (Rejecter - Accepter)** | | | | | | |
| no significant results |  |  |  |  |  |  |

* The '*' mark indicates the brain regions whose activations were statistically significant at the significance of p<0.05 (peak-level or cluster-level FWE corrected). The anatomical labelling was based on AAL3 and the peak coordinates which could not be classified by AAL3 were not reported.

**Supplementary Table 4. The peak coordinates of the repeated-measure flexible-factorial ANOVA results**

| **Anatomical description** | **Cluster-level P_FWE_** | **K** | **F** | **x** | **y** | **z** |
| --- | --- | --- | --- | --- | --- | --- |
| **Outcome onset: Type main effect** | | | | | | |
| **Fusiform gyrus/Cerebellum, R** | **0.006*** | 273 | 18.31 | 26 | -48 | -20 |
|  |  |  | 16.87 | 24 | -42 | -12 |
|  |  |  | 15.57 | 12 | -50 | -18 |
| Superior temporal gyrus, R | 0.929 | 21 | 13.72 | 64 | -40 | 12 |
| **Outcome onset: Time main effect** | | | | | | |
| **Lingual gyrus/Fusiform gyrus** | **0.000*** | 3694 | **62.5*** | -12 | -78 | -10 |
|  |  |  | **59.88*** | 26 | -72 | -14 |
|  |  |  | **57.51*** | 14 | -72 | -8 |
| **Dorsolateral prefrontal cortex/Superior temporal gyrus(temporal pole)** | **0.000*** | 1107 | **45.55*** | -42 | 30 | -18 |
|  |  |  | **29.58*** | -24 | 54 | 30 |
|  |  |  | **29.04*** | -48 | 20 | -12 |
| **Superior temporal gyrus/middle frontal gyrus, R** | **0.000*** | 4422 | **43.02*** | 62 | -6 | 0 |
|  |  |  | **39.72*** | 66 | -20 | 14 |
|  |  |  | **37.71*** | 40 | 50 | 10 |
| **Anterior cingulate cortex/dorsomedial prefrontal cortex** | **0.000*** | 641 | **36.05*** | 0 | 32 | 30 |
|  |  |  | 16.8 | 2 | 22 | 18 |
|  |  |  | 15.68 | -2 | 48 | 44 |
| **Cerebellum, L** | **0.000*** | 719 | **32.64*** | -26 | -70 | -36 |
|  |  |  | **32.27*** | -40 | -74 | -40 |
|  |  |  | 18.85 | -20 | -84 | -34 |
|  | 0.284 | 84 | 21.09 | -28 | -52 | -22 |
|  |  |  | 14.38 | 48 | -12 | -6 |
|  |  |  | 15.07 | -24 | -34 | -32 |
|  | 0.969 | 15 | 14.09 | 0 | -80 | -26 |
|  | 0.806 | 33 | 14.02 | -10 | -60 | -16 |
|  |  |  | 13.76 | -18 | -62 | -20 |
| **Superior temporal gyrus, L** | **0.001*** | 357 | **31.84*** | -62 | -14 | 10 |
|  |  |  | 20.6 | -60 | -2 | 2 |
|  |  |  | 18.35 | -58 | -30 | 14 |
|  | 0.979 | 13 | 13.31 | -40 | -36 | 12 |
| Fusiform gyrus, R | 0.248 | 90 | **28.03*** | 28 | -36 | -22 |
| **Occipital gyrus, R** | **0.016*** | 217 | **27.6*** | 28 | -86 | 22 |
|  |  |  | 15.28 | 32 | -72 | 24 |
| **Putamen, L** | **0.001*** | 401 | **26.49*** | -12 | -6 | 12 |
|  |  |  | 18.69 | -2 | -4 | 12 |
|  |  |  | 16.67 | -26 | -4 | 2 |
| **Cerebellum, R** | **0.001*** | 367 | 25.24 | 38 | -74 | -42 |
|  |  |  | 22.87 | 30 | -76 | -36 |
|  |  |  | 17.06 | 22 | -64 | -34 |
|  | 0.974 | 14 | 12.43 | 40 | -56 | -36 |
| **Middle occipital gyrus/Angular gyrus, L** | **0.004*** | 291 | 24.07 | -54 | -60 | 38 |
|  |  |  | 21.33 | -46 | -74 | 22 |
|  |  |  | 21.08 | -48 | -70 | 34 |
| **Precuneus/Cuneus, L** | **0.001*** | 363 | 21.77 | 2 | -70 | 38 |
|  |  |  | 21.06 | -2 | -56 | 38 |
|  |  |  | 18.14 | 2 | -82 | 24 |
| Calcarine/Middle occipital gyrus, R | 0.184 | 103 | 21.56 | 22 | -96 | 4 |
|  |  |  | 17.17 | 26 | -88 | 2 |
| Pregenual anterior cingulare cortex, L | 0.107 | 127 | 21.47 | -8 | 44 | 16 |
|  |  |  | 14.59 | -6 | 48 | 4 |
| Middle occipital gyrus, L | 0.487 | 60 | 19.29 | -26 | -64 | 34 |
| **Superior frontal gyrus, L** | **0.01*** | 241 | 18.74 | -14 | 24 | 62 |
| Middle frontal gyrus, L |  |  | 18.06 | -36 | 18 | 56 |
| Supplementary motor area, L |  |  | 16.24 | -2 | 18 | 64 |
|  | 0.912 | 23 | 18.48 | -34 | -48 | -42 |
| Middle cingulate cortex, L | 0.125 | 120 | 17.51 | -2 | -14 | 40 |
|  |  |  | 15.1 | -4 | -4 | 34 |
| Inferior temporal gyrus, L | 0.964 | 16 | 16.94 | -62 | -32 | -18 |
| Middle temporal gyrus, L | 0.4 | 69 | 16.44 | -60 | -10 | -18 |
| Precentral gyrus, L | 0.863 | 28 | 16.34 | -38 | -6 | 48 |
| Inferior pariental gyrus, L | 0.884 | 26 | 16.14 | -28 | -50 | 44 |
| Ventral Striatum, R | 0.945 | 19 | 15.11 | 12 | 14 | -6 |
| Precentral gyrus, R | 0.83 | 31 | 15.03 | 36 | -2 | 50 |
|  | 0.758 | 37 | 14.7 | 38 | 4 | 34 |
| Lingual gyrus, R | 0.818 | 32 | 14.13 | 12 | -44 | 2 |
| **Outcome onset: Type * Time effect** | | | | | | |
| Anterior/mid cingulate cortex, L | 0.072 | 145 | 19.71 | -10 | 28 | 26 |
|  |  |  | 16.19 | -8 | 20 | 32 |
| Superior/middle temporal gyrus, L | 0.391 | 70 | 18.64 | -50 | -12 | -12 |
|  |  |  | 11.69 | -48 | -14 | -4 |
| Temporal pole/Insula, R | 0.635 | 47 | 15.85 | 48 | 6 | -18 |
|  |  |  | 15.41 | 42 | 10 | -10 |
| Cerebellum, L | 0.894 | 25 | 15.75 | -26 | -42 | -24 |
| Superior frontal gyrus, medial orbital(ventromedial prefrontal cortex) | 0.979 | 13 | 14.04 | 6 | 46 | -6 |
| Middle cingulate cortex, R | 0.852 | 29 | 13.66 | 4 | 10 | 36 |
|  |  |  | 12.67 | 4 | 20 | 32 |
| **Choice onset: Role main effect** | | | | | | |
| **Middle cingulate gyrus** | **0.005*** | 347 | 21.39 | 4 | -36 | 32 |
|  |  |  | 18.57 | 2 | -40 | 48 |
|  |  |  | 16.07 | 6 | -38 | 40 |
| Precentral gyrus, L | 0.508 | 66 | 19.34 | -58 | 2 | 20 |
| Calcarine gyrus, R | 0.144 | 137 | 16.9 | 6 | -56 | 10 |
| Middle cingulate gyrus | 0.149 | 135 | 16.77 | 2 | 4 | 34 |
|  |  |  | 15.19 | -4 | -4 | 36 |
|  |  |  | 11.46 | 6 | -8 | 36 |
| IFG pars orbitalis, R | 0.982 | 10 | 16.12 | 50 | 22 | -8 |
| Superior frontal gyrus (dorsolateral prerfrontal gyrus) | 0.924 | 21 | 15.31 | 28 | 46 | 38 |
| Precuneus, L | 0.804 | 35 | 15.17 | -10 | -60 | 18 |
| Supplementary motor area, R | 0.598 | 56 | 14.94 | 12 | -10 | 52 |
| Inferior occipital gyrus, L | 0.755 | 40 | 14.08 | -40 | -68 | -8 |
|  |  |  | 12.45 | -46 | -76 | -8 |
| IFG, triangular part, R | 0.937 | 19 | 13.78 | 48 | 36 | 8 |
| Precentral gyrus, L | 0.937 | 19 | 13.77 | -52 | 0 | 34 |
| Crebellum, L | 0.931 | 20 | 13.51 | -22 | -38 | -20 |
| Temporal pole: superior temporal gyrus, L | 0.965 | 14 | 13.46 | -30 | 12 | -26 |
| Middle frontal gyrus | 0.982 | 10 | 13.12 | -26 | 34 | 26 |
|  | 0.982 | 10 | 12.66 | 32 | 44 | 30 |
| **Choice onset: Type main effect** | | | | | | |
| Insula/Temporal pole, L | 0.221 | 113 | 17.24 | -32 | 20 | -8 |
|  |  |  | 13.2 | -42 | 14 | -16 |
| Middle frontal gyrus, R | 0.931 | 20 | 13.99 | 34 | 18 | 48 |
| Supplementary motor area, R | 0.965 | 14 | 13.2 | 10 | 24 | 56 |
| **Choice onset: Role * Type interaction effect** | | | | | | |
| Rostromedial prefrontal cortex | 0.113 | 151 | 21.14 | 0 | 54 | 0 |
| Middle temporal gyrus, L | 0.965 | 14 | 14.02 | -62 | -32 | -16 |
| Superior frontal gyrus, R | 0.965 | 14 | 12.55 | 16 | 56 | 24 |

* The '*' mark indicates the brain regions whose activations were statistically significant at the significance of p<0.05 (peak-level or cluster-level FWE corrected). The anatomical labelling was based on AAL3 and the peak coordinates which could not be classified by AAL3 were not reported.
